# Revisiting Disinfection Byproducts with Supercritical Fluid Chromatography-High Resolution-Mass Spectrometry: Identification of Novel Halogenated Sulfonic Acids in Disinfected Drinking Water

**DOI:** 10.1021/acs.est.2c05536

**Published:** 2023-02-20

**Authors:** Maolida Nihemaiti, Maik Icker, Bettina Seiwert, Thorsten Reemtsma

**Affiliations:** † Department of Analytical Chemistry, Helmholtz Centre for Environmental Research - UFZ, Permoserstrasse 15, 04318 Leipzig, Germany; ‡ Institute of Organic Chemistry, University of Leipzig, Johannisallee 29, 04103 Leipzig, Germany; § Institute of Analytical Chemistry, University of Leipzig, Linnéstrasse 3, 04103 Leipzig, Germany

**Keywords:** DBPs, chlorination, drinking water treatment plant, tap water, swimming pool, SFC, HRMS, nontarget screening

## Abstract

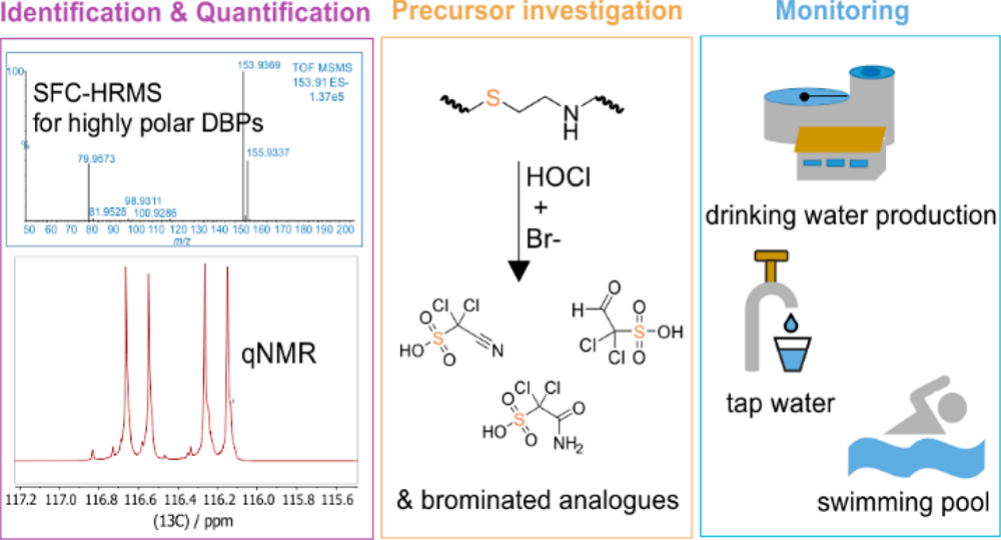

High resolution mass spectrometry (HRMS) coupled to either gas chromatography or reversed-phase liquid chromatography is the generic method to identify unknown disinfection byproducts (DBPs) but can easily overlook their highly polar fractions. In this study, we applied an alternative chromatographic separation method, supercritical fluid chromatography-HRMS, to characterize DBPs in disinfected water. In total, 15 DBPs were tentatively identified for the first time as haloacetonitrilesulfonic acids, haloacetamidesulfonic acids, and haloacetaldehydesulfonic acids. Cysteine, glutathione, and *p*-phenolsulfonic acid were found as precursors during lab-scale chlorination, with cysteine providing the highest yield. A mixture of the labeled analogues of these DBPs was prepared by chlorination of ^13^C_3_-^15^N-cysteine and analyzed using nuclear magnetic resonance spectroscopy for structural confirmation and quantification. A total of 6 drinking water treatment plants utilizing various source waters and treatment trains produced sulfonated DBPs upon disinfection. Those were widespread in the tap water of 8 cities across Europe, with estimated concentrations up to 50 and 800 ng/L for total haloacetonitrilesulfonic acids and haloacetaldehydesulfonic acids, respectively. Up to 850 ng/L haloacetonitrilesulfonic acids were found in 3 public swimming pools. Considering the stronger toxicity of haloacetonitriles, haloacetamides, and haloacetaldehydes than the regulated DBPs, these newly found sulfonic acid derivatives may also pose a health risk.

## Abstract 2

Fifteen novel sulfonated disinfection byproducts were identified using supercritical fluid chromatography-high resolution mass spectrometry and widespread in drinking water treatment plant, tap water, and swimming pool samples across Europe.

## 1 Introduction

Disinfection of drinking water is an important step to prevent acute waterborne diseases. However, the chemical disinfectants (e.g., free chlorine, monochloramine) react with natural organic matter, anthropogenic compounds, bromide, and iodide to produce undesired disinfection byproducts (DBPs).^1,2^ Epidemiological studies suggested the association of DBP exposure with the risk of bladder cancer and birth defects in humans.^3,4^ Over 700 DBPs have been characterized so far.^5^ Of these, the (semi)volatile classes are quantified using gas chromatography–mass spectrometry (GC-MS), which accounts for less than 50% of total organic halogens (TOX) produced during chlorination.^6,7^ A small number of DBPs, such as trihalomethanes (THMs) and haloacetic acids (HAAs), are regulated. However, many unregulated DBPs (e.g., haloacetonitriles, haloacetamides, haloacetaldehydes) are more cytotoxic and genotoxic than the regulated THMs and HAAs.^8^ As a result, revealing the unknown DBPs has been the focus of many studies aiming to find and to identify the toxic potencies in disinfected water.

GC or reversed-phase liquid chromatography (RPLC) coupled to (high resolution) mass spectrometry, (HR)MS, has been increasingly applied in recent years for the detection of novel DBPs.^9^ Such examples include the successful identification of halogenated phenolic substances,^10^ nitrogenous heterocyclic compounds,^11,12^ and haloquinone chloroimides^13^ in chlorinated or chloraminated water. However, the generic GC or RPLC-MS methods can result in significant analytical gaps by overlooking the very polar fractions of DBPs, which are nonvolatile for GC analysis and hardly retainable on RPLC stationary phases (e.g., C18 column).^14^ Therefore, the application of alternative chromatographic separation methods, such as hydrophilic interaction chromatography (HILIC) or supercritical fluid chromatography (SFC), can be useful to narrow such analytical gaps in analyzing polar substances.^14^ For instance, Zahn et al. recently applied HILIC-HRMS to identify halomethanesulfonic acids (HMSAs) as a new class of polar DBPs in disinfected water.^15,16^

SFC has a unique mobile phase comprising supercritical CO_2_ (nonpolar) and polar modifier (e.g., methanol) and is compatible with both polar and nonpolar stationary phases. These features allow SFC to analyze a large number of analytes of a wide polarity range.^17^ SFC-MS has been widely applied in the pharmaceutical industry, metabolomics analysis, and food science.^18^ It is receiving increasing attention for the characterization of persistent and mobile chemicals in aquatic environments.^19,20^ SFC separation was reported to be superior to RPLC in terms of peak shapes and retention of PM chemicals, thus considerably facilitating signal detection and integration.^21^ A recent study on wastewater ozonation demonstrated that SFC-HRMS was able to detect those ozonation products that are extremely hydrophilic and persistent to post-treatments but have been overlooked during the generic RPLC-HRMS measurements.^22^

In this study, SFC-HRMS was applied to revisit DBPs in disinfected water from drinking water treatment plants (DWTPs), tap water, and swimming pools. A number of 15 novel halogenated sulfonic acids were tentatively identified for the first time. Detailed lab-scale chlorination experiments were conducted to investigate their potential precursors and formation mechanisms. Despite the lack of analytical standards, chlorination of the precursor compound (i.e., cysteine) combined with nuclear magnetic resonance spectroscopy (NMR) and SFC-HRMS analysis provided an alternative approach for structural confirmation and quantification of newly found DBPs in water samples.

## 2 Materials and Methods

### 2.1 Chemicals

All chemicals were of analytical grade and used as-received without further purification. Carbon dioxide Premium (4.5) was used for SFC. Methanol, acetonitrile, and formic acid were provided by Biosolve (Valkenswaard, Netherlands). A sodium hypochlorite solution (6–14%) was provided by Th. Geyer GmbH (Hamburg, Germany). Disodium hydrogen phosphate (≥99%), potassium dihydrogen phosphate (≥99%), cysteine (≥99%), and glutathione (99%) were purchased from Merck (Darmstadt, Germany). *p*-Phenolsulfonic acid hydrate (85%) was supplied by abcr GmbH (Karlsruhe, Germany). Suwannee River Fulvic Acid (SRFA) was purchased from the International Humic Substances Society (SRFA II; 2S101H). The in-house synthesized and purified standards of chloromethanesulfonic acid (ClMSA), bromomethanesulfonic acid (BrMSA), dichloromethanesulfonic acid (Cl_2_MSA), bromochloromethanesulfonic acid (BrClMSA), and dibromomethanesulfonic acid (Br_2_MSA) were offered by D. Zahn.^16^ Ultrapure water was obtained from a Merck Milli-Q Integral 5 system (Darmstadt, Germany).

### 2.2 Water Samples

Tap water samples from 8 cities in Europe (Prague, Venice, Sardinia, Marseille, Leipzig, Brussels, Stockholm, and Uppsala) were collected during summer 2021. Samples from 3 public swimming pools in Germany were taken in February 2022. Grab samples from 6 DWTPs before and after disinfection, including DWTP 1 and 2 in Germany (5 sets each of repeated samples every 2 weeks), DWTP 3, 4, and 5 in Hungary (3 sets each of repeated samples every 2 months), and DWTP 6 in Spain (1 set of sample), were collected during January-November 2021 (sampling details are given below). DWTP 1 uses the mixture of groundwater and river bank filtrate as source water. The treatment trains include aeration, gravel filtration, and chlorine gas disinfection. DWTP 2 uses river bank filtrate and utilizes aeration, flocculation, gravel filtration, and chlorine dioxide disinfection. DWTP 3, 4, and 5 use river bank filtrate. In DWTP 3 and 4, the raw water is directly disinfected using sodium hypochlorite and chlorine gas, respectively, without additional treatment. In DWTP 5, the raw water is treated by ozone and sand filtration for iron and manganese removal, followed by chlorine gas disinfection. The disinfected water from DWTP 3, 4, and 5 was used to provide drinking water to a city in Hungary. Two sets of additional samples from 2 entry points to the distribution system and 2 drinking water storage reservoirs within this city were collected in September and November 2021. DWTP 6 uses surface water, which is treated through flocculation and filtration and by chlorine gas, as the final step.

Samples were collected in prewashed 100 mL borosilicate brown glass bottles, transported to the lab in the thermobox (10–12 °C), and enriched within 24 h of collection (see section 2.3). The residual chlorine in all disinfected samples was measured online or using a portable device (Pocket Colorimeter II, HACH) during sampling, which was in the range of ∼0.2–0.3 mg/L as Cl_2_ except for DWTP 6 (i.e., ∼0.5–1.0 mg/L as Cl_2_). Lab tests suggested that some of the novel halogenated sulfonic acids are not stable in the presence of a quenching reagent (e.g., ascorbic acid, Supporting Information, Figure S1); thus no additional chemical was added to quench the residual chlorine during sampling. A control sample prepared with 100 mL of ultrapure water spiked with 0.3 mg/L chlorine was preserved for 24 h, enriched, and analyzed following the same procedure as water samples. Results indicated that no contamination can occur during sample processing and analysis due to the potential presence of a trace amount of chlorine in the disinfected samples. Detailed information on sampling dates and water parameters is given in Table S1.

### 2.3 Sample Enrichment

Water samples were enriched using a freeze-dryer (Alpha1-4, Christ, Germany). A 40 mL aliquot of each sample was transferred into a 50 mL falcon tube and prefrozen at −20 °C overnight, followed by freeze-drying at 15 °C and 1.65 mbar for 30 h until dryness. The residue was reconstituted in 400 μL of acetonitrile and water (90:10, v:v), transferred into an Eppendorf tube, and then centrifuged at 13000 min^–1^ for 10 min. The supernatant was then transferred into glass vials and stored at −20 °C until analysis. An ultrapure water blank was prepared following the same procedure.

### 2.4 Lab-Scale Chlorination Experiments

Chlorination experiments were conducted in 10 mM phosphate buffer (pH 7) in amber glass bottles at room temperature (23 ± 2 °C) for 5–48 h. The commercial solution of sodium hypochlorite was standardized by measuring the absorbance of the hypochlorite anion at 292 nm (ε = 362 M^–1^ cm^–1^).^23^ The chlorination of cysteine, glutathione, and *p*-phenolsulfonic acid was conducted individually by applying 250 μM each compound and 0.5–2.5 mM initial chlorine. The chlorination of the SRFA extract (5 mg/L dissolved organic carbon, DOC) was performed by applying 5 mg/L as Cl_2_ of initial chlorine. The residual chlorine was measured using the DPD colorimetric method^24^ and quenched using a 2-fold molar excess of Na_2_S_2_O_3_. The chlorinated solutions of cysteine and glutathione were directly analyzed on SFC-QTOF without further enrichment, while the chlorinated *p*-phenolsulfonic acid and SRFA were analyzed after freeze-drying enrichment.

### 2.5 SFC-QTOF Analysis

Samples were analyzed using an ACQUITY UPC^2^ system coupled with a Synapt GS2 QTOF (Waters, Eschborn, Germany). Compounds were separated on a BEH column (3.0 mm × 100 mm, 1.7 μm, Waters, Eschborn, Germany) coupled at 55 °C with a flow rate of 1.5 mL/min and an injection volume of 5 μL. The mobile phase comprised (A) CO_2_ and (B) methanol/water cosolvent (95/5 by volume) containing 10 mM ammonium formate. The gradient was applied as follows: 0–0.5 min, 1% B; 9–12.5 min, 50% B; 12.6–15 min, 1% B. A methanol/water (90/10 by volume) makeup flow with 0.1% formic acid was used to transfer the column effluent into the mass spectrometer at 0.3 mL/min. Samples were analyzed in negative electrospray ionization (ESI) mode. A lock-spray containing leucine enkephalin was continuously infused during measurement. The source settings include capillary voltage of −2 kV, source temperature at 140 °C, and desolvation temperature at 550 °C. The sampling cone voltage and source offset were set as 20 and 40 V, respectively. Nitrogen and argon were used as cone and collision gases, respectively. The desolvation gas flow was 950 L/h. The data was recorded in centroid mode with a 0.08-s scan time over the mass range of *m*/*z* 50–1200 (resolution approximately 20000). The MS^E^ acquisition was performed to simultaneously collect two data sets: a low-collision-energy scan to obtain parent ion information and an elevated-collision-energy scan (15–40 eV) to get all fragment ions. The MS/MS spectra of the newly identified sulfonated DBPs in the chlorinated cysteine solution were also recorded using a collision energy ramp of 15 to 40 eV.

Data was processed using Waters MassLynx, MarkerLynx, and TargetLynx software. Exact molecular formulas of unknown DBPs were assigned based on the combination of elements C_0–20_, H_0–100_, N_0–5_, O_0–10_, P_0–2_, S_0–2_, I_0–4_, Br_0–4_, Cl_0–4_, and Na_0–2_ within the mass tolerance of 5 ppm. Molecular formulas with Cl and Br were confirmed manually using a simulated isotope model.

### 2.6 NMR Analysis and Quantification of DBPs

The ^13^C_3_-^15^N labeled cysteine (1 mM) was chlorinated for 48 h by applying 5 mM initial chlorine in 200 mL of the phosphate buffer solution (10 mM, pH 7). The solution was then enriched by freeze-drying following the procedure mentioned in section 2.3 and reconstituted in acetonitrile-d3 and water-d2 (9:1, v-v). For NMR analyses, an aliquot (230 μL) of this extract was spiked with 100 μL of ^13^C-urea (4.23 mM) and 2 mg of Cr(acac)_3_. The 100 MHz ^13^C NMR spectra were recorded using a 400 MHz Bruker AVIII HD spectrometer with a 30° flip angle and a repetition delay of 35 s (^1^H decoupling only during acquisition, pulse sequence: zgig30). The structures of the ^13^C_2_-^15^N labeled mono- and dichloroacetonitrilesulfonic acids (ClANSA and Cl_2_ANSA) and the ^13^C_2_ labeled dichloroacetaldehydesulfonic acid (Cl_2_AcAlSA) were further confirmed by their respective chemical shifts (^1^H, ^13^C, and ^15^N NMR spectra) and 2D NMR correlation spectra. Their concentrations were estimated based on their relative abundances to ^13^C-urea (used as the internal quantification standard) in ^13^C NMR spectra.

Another aliquot (10 μL) of this extract was used to make a serial dilution in acetonitrile and water (9:1, v:v). Forty μL aliquots of these diluted solutions were then spiked into the nondisinfected drinking water samples (40 mL each) from DWTPs 1 and 2. The processed calibration curves of the above compounds were made on SFC-QTOF following the freeze-drying enrichment of these spiked samples, which were then used to estimate the concentration of novel sulfonated DBPs in water samples. The concentration of brominated DBPs was estimated based on their chlorinated analogues. The limit of quantification (LOQ) for ClANSA and Cl_2_ANSA was 20 ng/L, and it was 40 ng/L for Cl_2_AcAlSA on SFC-QTOF.

## 3 Results and Discussion

### 3.1 Tentative Identification of Novel Sulfonated DBPs in Disinfected Drinking Water

Drinking water samples before and after chlorination from DWTP 1 were screened using SFC-QTOF. Several new peaks in negative ionization mode were observed in chlorinated water (Figure S2, base peak chromatogram), which were absent before disinfection and thus considered as DBPs. A total of 15 compounds were tentatively identified as the sulfonic acid derivatives of haloacetonitriles, haloacetamides, and haloacetaldehydes (Table 1). Their structures were proposed based on the exact masses and fragmentation patterns obtained from HRMS up to identification level 2 (Figures S3–S17, extracted ion chromatogram and MS/MS spectrum).^25^ Of these, the identification confidence for ClANSA, Cl_2_ANSA, and Cl_2_AcAlSA was further improved to level 1 by NMR analysis (see section 3.3). These sulfonated DBPs were also observed during SFC-QTOF screening of the disinfected water samples from other DWTPs, drinking water distribution systems, tap water, and swimming pools (see section 3.4).

**Table 1:**
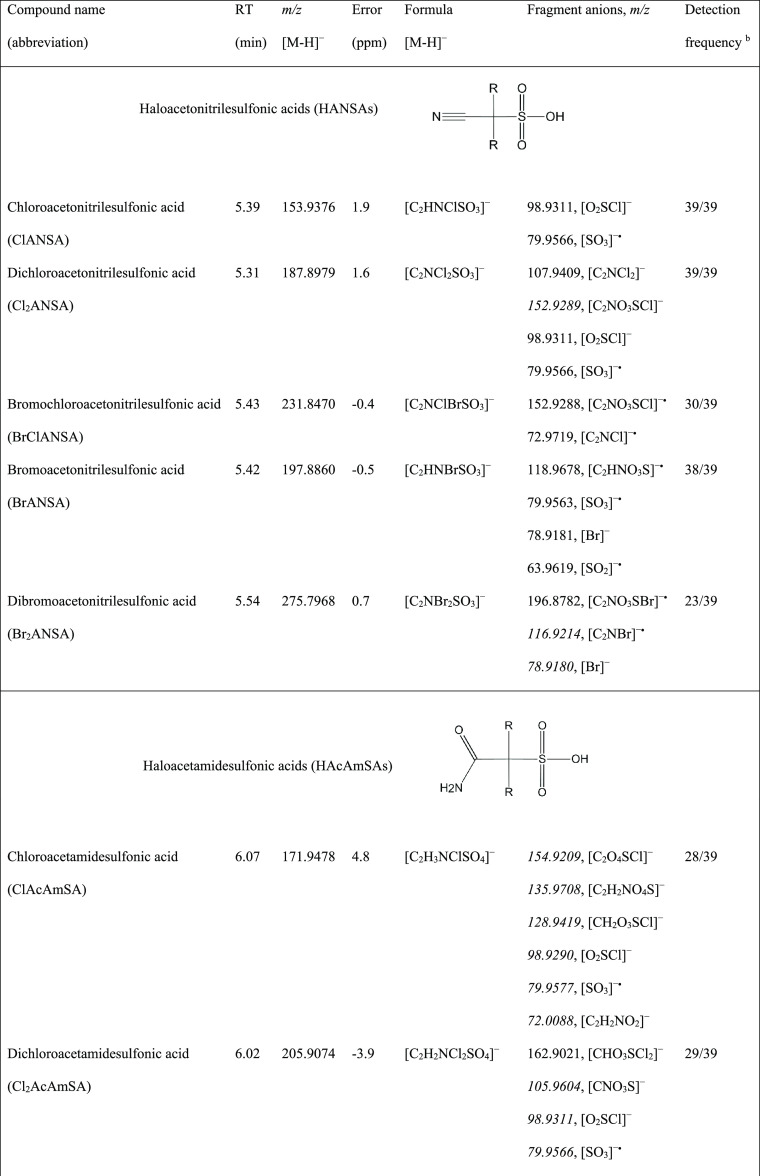

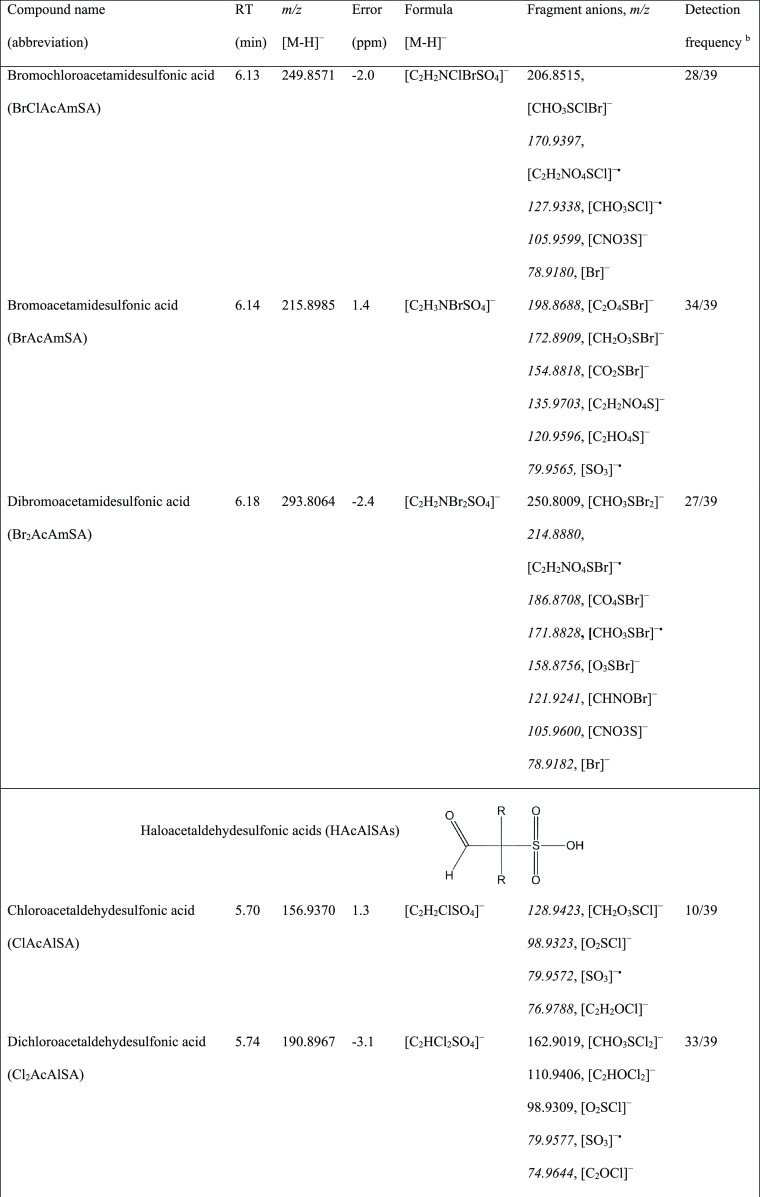

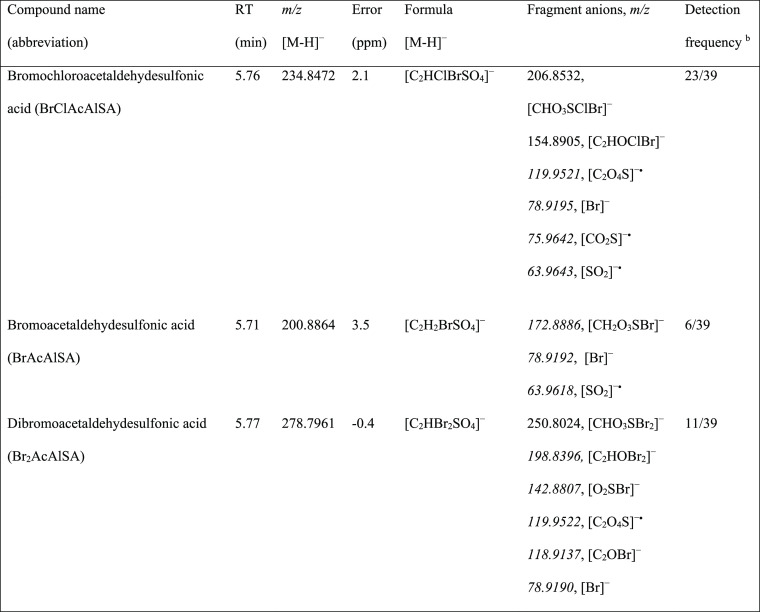
Identity, Retention Time, and Mass Spectrometric Data of the Novel Sulfonated DBPs Detected from the Disinfected Water Samples (from DWTPs, Drinking Water Distribution Systems, Tap Water, or Swimming Pool Water) and Lab-Scale Chlorination of Cysteine by SFC-HRMS in This Study^a^

a Compounds were identified with SFC-QTOF in the ESI negative mode. The extracted ion chromatogram and MS/MS spectra of compounds are displayed in Figures S3–S17. R in chemical structures represents H, Cl, or Br. Fragment ions highlighted in *italics* were only detected from high concentration in the chlorinated cysteine solution.

b Detection frequency in a total of 39 disinfected water samples collected from tap water (8), DWTPs (20), inlet to drinking water distribution systems (4), drinking water storage reservoirs (4), and public swimming pools (3).

The compound at retention time (RT) of 5.31 min (Table 1) contains isotopologues at *m*/*z* 188/190/192 with an isotopic abundance ratio (9:6:1) characteristic for the presence of two chlorine atoms. The accurate mass *m*/*z* 187.8986 corresponds with the molecular ion formula of [C_2_NCl_2_SO_3_]^−^ (mass difference: 1.6 ppm). Its mass spectrum exhibits fragment isotopologues at *m*/*z* 108/110/112, consistent with the elemental formula of [C_2_NCl_2_]^−^ (*m*/*z* 107.9409, 0.9 ppm), and at *m*/*z* 79.9566, corresponding to [SO_3_]^−**•**^ (−2.5 ppm), an indicator of sulfonic acids.^26^ The isotopologues at *m*/*z* 99/101 (*m*/*z* 98.9311, [O_2_SCl]^−^, 3 ppm) were also found, which were likely produced by rearrangement during fragmentation. Therefore, this compound was tentatively identified as dichloroacetonitrilesulfonic acid (Cl_2_ANSA).

The compound at RT = 5.42 min contains predominant isotopologues at *m*/*z* 198/200 with isotopic abundance (1:1), indicating that this compound might contain one bromine atom. This was further confirmed by the presence of fragment isotopologues at *m*/*z* 79/81 for [Br]^−^ (−2.5 ppm). The accurate mass *m*/*z* 197.8860 supported a molecular ion formula of [C_2_HNBrSO_3_]^−^ (−0.5 ppm). Furthermore, the radical anions [SO_3_]^−**•**^ and [SO_2_]^−**•**^ as fragment ions reveal this compound as a sulfonic acid.^26^ Accordingly, it was proposed as bromoacetonitrilesulfonic acid (BrANSA). A similar method was applied for the tentative identification of compounds at RT = 5.39, 5.43, and 5.54, which were proposed as the chloro (ClANSA), bromochloro (BrClANSA), and dibromo (Br_2_ANSA) analogues, respectively, of Cl_2_ANSA and BrANSA (Table 1).

The compound at RT = 6.07 min has dominant isotopologues at *m*/*z* 172/174, indicative of the presence of one chlorine atom. Its accurate mass *m*/*z* 171.9478 is in accordance with a molecular ion formula of [C_2_H_3_NClSO_4_]^−^ (−4.8 ppm). Its mass spectrum shows fragments at *m*/*z* 155/157 ([C_2_O_4_SCl]^−^) and *m*/*z* 129/131 ([CH_2_O_3_SCl]^−^) corresponding to the loss of either an −NH_3_ group or a −CHNO group, respectively, from the parent ion, revealing the presence of an amide group. Accordingly, this compound was proposed as chloroacetamidesulfonic acid (ClAcAmSA). Due to the low intensity of the parent ion, the radical ion [SO_3_]^−**•**^ was not visible as a fragment of this compound in drinking water samples. Nevertheless, during lab-scale chlorination of cysteine (see section 3.2), fragments at *m*/*z* 79.9566 ([SO_3_]^−**•**^) and 98.9311 ([O_2_SCl]^−^) were found, which was in accordance with the proposal for sulfonic acid. The dichloro (Cl_2_AcAmSA), bromochloro (BrClAcAmSA), bromo (BrAcAmSA), and dibromo (Br_2_AcAmSA) analogues of ClAcAmSA were also observed in chlorinated drinking water (Table 1).

The third group of novel sulfonated DBPs was proposed as haloacetaldehydesulfonic acids (HAcAlSAs). The compound at RT = 5.74 min is a dichlorinated compound with isotopologues at *m*/*z* 191/193/195, accurate mass of which supports a molecular formula of [C_2_HCl_2_SO_4_]^−^ (−3.1 ppm). It exhibits fragment ions at *m*/*z* 163/165/167 ([CHO_3_SCl_2_]^−^) following the loss of a −CO group and fragments at *m*/*z* 111/113/115 ([C_2_HOCl_2_]^−^) corresponding to the loss of a −SO_3_ group. This compound also contains the radical ion [SO_3_]^−**•**^ as a fragment at *m*/*z* 79.9577. Therefore, it was tentatively identified as dichloroacetaldehydesulfonic acid (Cl_2_AcAlSA). The bromochloro (BrClAcAlSA) and dibromo (Br_2_AcAlSA) analogues of Cl_2_AcAlSA were observed from the disinfected water samples, and all shared a similar pattern in mass spectra by losing a −CO and an −SO_3_ group during fragmentation (Table 1). The chloro (ClAcAlSA) and bromo (BrAcAlSA) analogues were also found in some samples during suspect screening but with much lower signal intensities.

### 3.2 Formation of Novel Sulfonated DBPs from the Chlorination of Cysteine and Other Precursors

Previous studies reported the presence of various sulfonated DBPs in disinfected water, such as iodotrihydroxybenzenesulfonic acids produced from the chlorination of saline wastewater,^27^ tribromoethenesulfonate detected from chlorinated ballast water,^28^ and HMSAs detected from chlorinated drinking water.^15,16^ However, little is known about the precursors and formation mechanisms of these sulfonated DBPs, including those 15 newly found in this study.

Nitrogen-containing organic compounds (e.g., amino acids, peptides, proteins) are ubiquitous in surface water and important precursors of nitrogenous DBPs.^29^ The amino acid cysteine, which has a thiol group and a primary amine moiety, was investigated as a model precursor of the sulfonated DBPs found in this study. The formation potential of the regulated and known DBPs from chlorination or chloramination of cysteine has been studied,^30−32^ but the formation of sulfonated DBPs has not been recognized likely due to the lack of appropriate analytical methods for these highly polar DBPs. Chlorination of cysteine was conducted in phosphate buffer at pH 7 by applying 250 μM cysteine and three different chlorine doses (0.5, 1.25, and 2.5 mM). High concentrations of cysteine and chlorine were applied here to maximize DBP formation for better signal intensity to support identification by SFC-QTOF. After 5 h of reaction, chlorine was fully consumed when 0.5 and 1.25 mM initial chlorine was applied, whereas 0.7 mM chlorine was left in the case of 2.5 mM initial chlorine. All chlorinated DBPs in Table 1 were detected. HANSAs (i.e., ClANSA, Cl_2_ANSA) were predominantly produced (based on signal intensity), followed by HAcAlSAs (i.e., ClAcAlSA, Cl_2_AcAlSA) and HAcAmSAs (i.e., ClAcAmSAs, Cl_2_AcAmSAs) (Figure S18). The monochlorinated analogues appeared as intermediates by reaching the highest intensity at 1.25 mM initial chlorine, while the dichlorinated analogues increased with an increasing chlorine dose (Figure S18), indicating that more chlorine substitution occurs with higher chlorine exposure.

An additional experiment was carried out by applying 250 μM cysteine, 1.25 mM chlorine, and 500 μM bromide. A high concentration of bromide was also chosen to maximize the formation of brominated DBPs to obtain better signal intensity on SFC-QTOF. All bromochloro, monobromo, and dibromo analogues in Table 1 were detectable after 5 h of reaction, revealing the incorporation of bromine into DBPs in the presence of bromide ion (Figure S19). Moreover, the formation of Cl_2_MSA, BrClMSA, and Br_2_MSA was also detected (Figure S19), suggesting that cysteine can also be a precursor of HMSAs previously found from disinfected drinking water.^15,16^ However, the intensities of HMSAs were much lower compared to their HANSA, HAcAmSA, and HAcAlSA analogues found in this study (Figures S18 and S19).

**Scheme 1:**
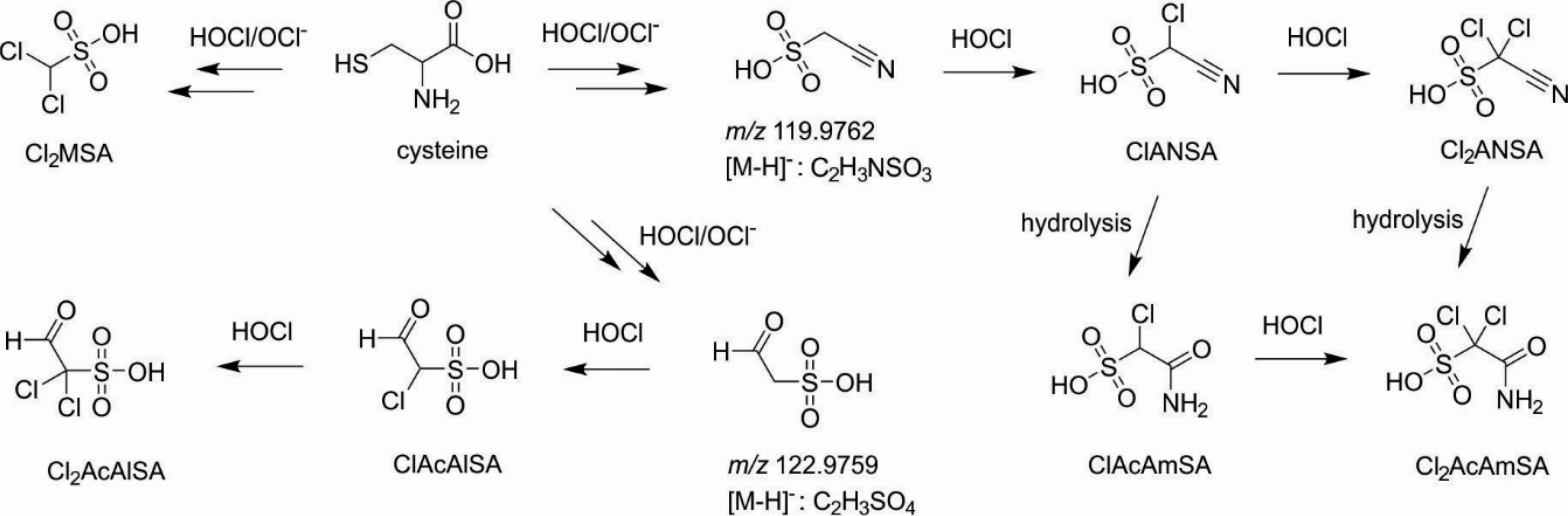
Proposed Formation Pathways of Sulfonated DBPs from the Chlorination of Cysteine Cl_2_MSA - dichloromethanesulfonic acid, ClANSA - chloroacetonitrilesulfonic acid, Cl_2_ANSA - dichloroacetonitrilesulfonic acid, ClAcAlSA - chloroacetaldehydesulfonic acid, Cl_2_AcAlSA - dichloroacetaldehydesulfonic acid, ClAcAmSA - chloroacetamidesulfonic acid, Cl_2_AcAmSA - dichloroacetamidesulfonic acid.

The formation pathways of sulfonated DBPs from the chlorination of cysteine are proposed in Scheme 1. It was reported that HOCl reacts with cysteine approximately 2 orders of magnitude faster than with nonsulfur containing amino acids.^33,34^ Thus, the primary chlorine attack is expected to occur on the reduced sulfur moiety, followed by the amine group.^33−35^ Both HOCl and ClO^–^ are highly reactive with the thiol group to produce sulfonic acid via sulfenyl chloride as the intermediate.^34,36^ Moreover, chlorine is highly reactive with the primary amine in α-amino acids to produce −RNHCl and −RNCl_2_, which ultimately form aldehyde and nitrile.^37^ In this study, two intermediates were observed following the chlorination of thiol and amine functional groups of cysteine (Scheme 1), which were tentatively identified as acetonitrilesulfonic acid (i.e., *m*/*z* 119.9762, [C_2_H_3_NSO_3_]^−^, mass spectrum in Figure S20) and acetaldehydesulfonic acid (i.e., *m*/*z* 122.9759, [C_2_H_3_SO_4_]^−^, mass spectrum in Figure S21). Interestingly, both intermediates were also detected from the disinfected water samples in this study (extracted ion chromatogram in Figures S20 and S21). Further chlorination of acetonitrilesulfonic acid can produce its mono- and dichloro substituted products: ClANSA and Cl_2_ANSA. Haloacetonitriles are well-known to hydrolyze into their corresponding haloacetamides.^38,39^ Therefore, the subsequent hydrolysis of ClANSA and Cl_2_ANSA was proposed to produce the chlorinated acetamidesulfonic acids: ClAcAmSA and Cl_2_AcAmSA. The further chlorination of another intermediate, acetaldehydesulfonic acid (*m*/*z* 122.9759), can lead to the formation of mono- (ClAcAlSA) and dichloro- (Cl_2_AcAlSA) acetaldehydesulfonic acids. Furthermore, as a minor reaction pathway (based on the intensities of products on SFC-QTOF), the oxidation of the thiol group and the substitution of chlorine on a neighboring carbon can eventually produce Cl_2_MSA following the cleavage of molecule (Scheme 1).

The formation potentials of novel sulfonated DBPs were also investigated during the chlorination of glutathione, a tripeptide with cysteine residue (containing a thiol group), and *p*-phenolsulfonic acid (with a sulfonate group). This was based on an assumption that the sulfonate group in these DBPs might originate from the reduced sulfur moieties via oxidation or be generated from compounds already containing a sulfonate group. Results indicated that all chlorinated analogues in Table 1 can be produced from glutathione chlorination, whereas *p*-phenolsulfonic acid can produce the chlorinated acetaldehydesulfonic acids (ClAcAlSA and Cl_2_AcAlSA). However, the signal intensities of these products were much lower than those generated from cysteine under similar experimental conditions (Figure S22a). In addition, the formation of chlorinated analogues in Table 1 was also observed during the chlorination of an SRFA extract as a natural organic matter surrogate (Figure S22b). Overall results suggested that more than one precursor of novel sulfonated DBPs can be present in water matrices, which could generate this product spectrum to various degrees upon chlorination.

### 3.3 Structural Confirmation and Quantification Using NMR

Since analytical standards of the novel sulfonated DBPs are not commercially available, the mixture of their chlorinated analogues (Table 1) was generated by lab-scale chlorination of cysteine, which was then analyzed using NMR after freeze-drying enrichment. To get better signal intensity and to gain more structural information in NMR analysis, the ^13^C_3_-^15^N labeled cysteine was employed. The structures of ^13^C_2_-^15^N labeled ClANSA and Cl_2_ANSA, as well as ^13^C_2_ labeled Cl_2_AcAlSA, were confirmed by NMR spectroscopy (Figure S23, Table S2, and Text S1). Furthermore, their concentrations in this DBP mixture were estimated using ^13^C-urea as the internal quantification standard (i.e., 549 μM, 391 μM, 139 μM for ^13^C_2_-^15^N-ClANSA, ^13^C_2_-^15^N-Cl_2_ANSA, and ^13^C_2_-Cl_2_AcAlSA, respectively, Table S3 and Text S2). The low intensities of other chlorinated analogues did not allow their accurate identification and quantification.

Moreover, the water samples from DWTPs 1 and 2 prior to disinfection were spiked with various amounts of this mixture and analyzed using SFC-QTOF following freeze-drying. No clear trend was observed on the apparent recovery rates of ^13^C_2_-^15^N-ClANSA, ^13^C_2_-^15^N-Cl_2_ANSA, and ^13^C_2_-Cl_2_AcAlSA depending on the water matrix or spiked concentrations (Figure S24). Good recovery was obtained for HANSA analogues (i.e., average apparent recovery rate of 100 ± 10% for ^13^C_2_-^15^N-ClANSA and 99 ± 7% for ^13^C_2_-^15^N-Cl_2_ANSA), whereas ^13^C_2_-Cl_2_AcAlSA was incompletely recovered (43 ± 10%). Both sample preparation recovery and matrix effect could contribute to the variability in the apparent recovery of substances during enrichment and instrumental analysis. Matrix effected signal enhancement or suppression depending on the analyte type was reported previously during SFC-MS analysis of persistent and mobile chemicals.^21^ Except for the potential matrix effect during SFC-QTOF analysis, the poor stability of aldehyde compounds may also cause their low recovery during freeze-drying. Future studies can explore other enrichment options, such as by a weak anion exchange (WAX) cartridge; this may allow the strongly acidic sulfonated DBPs to be enriched but dissolved organic matter and much of inorganic salts to pass through.

### 3.4 Occurrence of Novel Sulfonated DBPs in DWTPs, Tap Water, and Swimming Pools

The processed calibration curves of ^13^C_2_-^15^N-ClANSA, ^13^C_2_-^15^N-Cl_2_ANSA, and ^13^C_2_-Cl_2_AcAlSA showed good linearity (*R*^2^ > 0.96) between spiked concentrations vs peak area on SFC-QTOF after sample enrichment (Figure S25), which were then applied to estimate the concentrations of novel sulfonated DBPs in water samples. The concentrations of brominated DBPs were estimated based on the processed calibration curves of their chlorinated analogues. Specifically, the calibration curve of ^13^C_2_-^15^N-ClANSA was used for ClANSA and BrANSA, ^13^C_2_-^15^N-Cl_2_ANSA was used for Cl_2_ANSA, BrClANSA, and Br_2_ANSA, and ^13^C_2_-Cl_2_AcAlSA was applied for Cl_2_AcAlSA, BrClAcAlSA, and Br_2_AcAlSA. ClAcAlSA, BrAcAlSA, and HAcAmSAs were not quantified due to the lack of any suitable halogenated reference compound.

#### DWTPs

**Figure 1:**
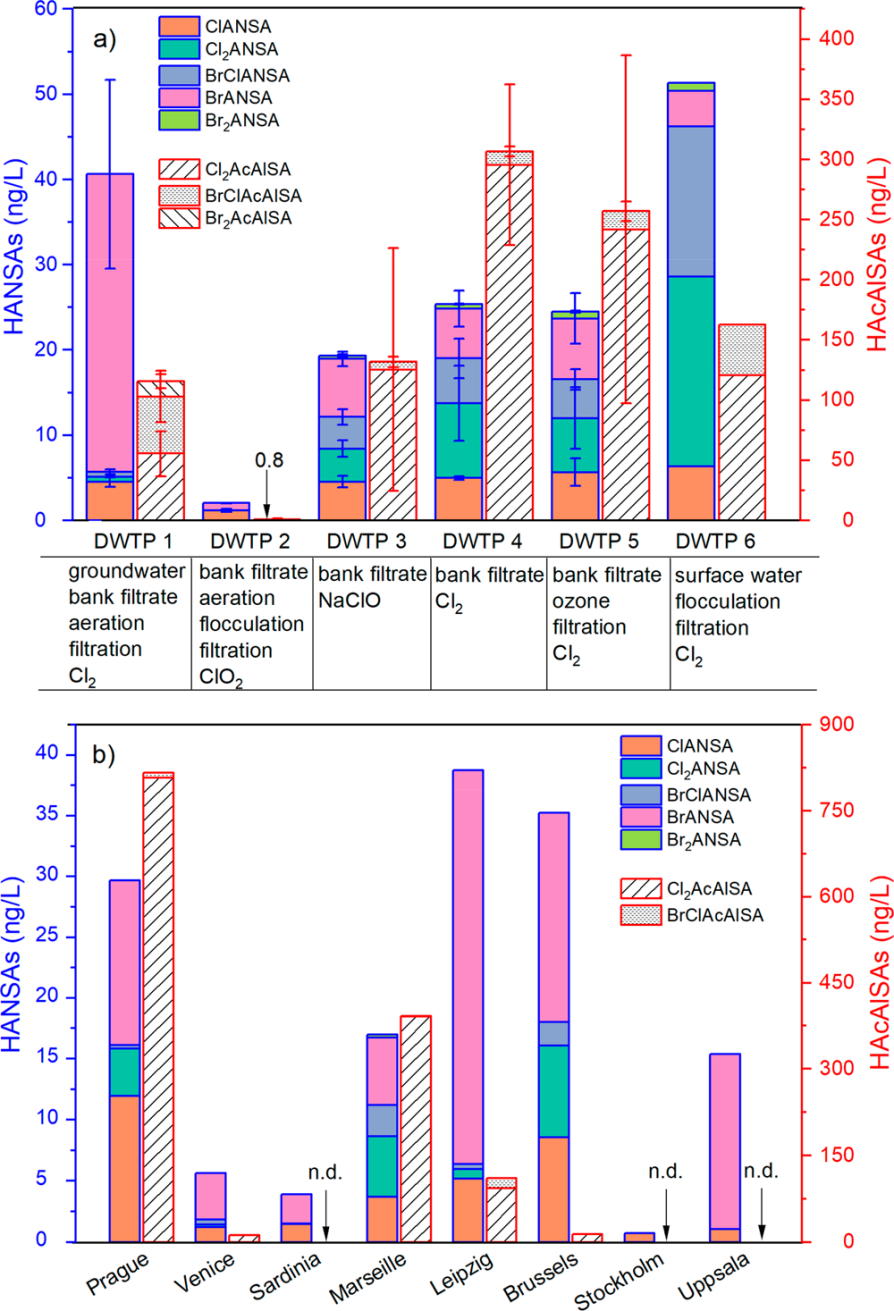
Estimated concentrations of HANSAs and HAcAlSAs (right *Y*-axis) in (a) the finished water from DWTPs and (b) tap water samples. The table below (a) shows the treatment processes utilized in each DWTP. The error bars in (a) represent the standard deviation of concentrations obtained during different sampling dates (five replicates for DWTP 1 and 2; three replicates for DWTP 3, 4, and 5). n.d. means nondetectable. ClANSA - chloroacetonitrilesulfonic acid, Cl_2_ANSA - dichloroacetonitrilesulfonic acid, BrClANSA - bromochloroacetonitrilesulfonic acid, BrANSA - bromoacetonitrilesulfonic acid, Br_2_ANSA - dibromoacetonitrilesulfonic acid, Cl_2_AcAlSA - dichloroacetaldehydesulfonic acid, BrClAcAlSA - bromochloroacetaldehydesulfonic acid, Br_2_AcAlSA - dibromoacetaldehydesulfonic acid.

Figure 1a shows the estimated concentrations of HANSAs and HAcAlSAs in the finished water samples from 6 different DWTPs. These newly identified sulfonated DBPs were formed to various degrees in all DWTPs regardless of the source water type and treatment trains, revealing the widespread presence of their precursors in groundwater, river bank filtrate, and surface water. The total concentrations of HANSAs were in the range of 2–50 ng/L. HAcAlSAs were present up to 300 ng/L, much higher than HANSAs (except for DWTP 2, ∼1 ng/L). Among these, Cl_2_AcAlSA predominated, followed by BrClAcAlSA and Br_2_AcAlSA. The sum of HANSAs and HAcAlSAs in DWTP 1 was 156 ng/L, which corresponded to 5% of its THMs (i.e., 3.1 μg/L). For DWTP 2, the THM was <0.5 μg/L, while for other DWTPs it is unknown.

DWTP 2 produces less DBPs than other plants despite its slightly higher TOC value (high precursor loading) in predisinfection water (∼2.5 mg/L vs 1–1.5 mg/L, Table S1). This can be explained by the application of ClO_2_ as a disinfectant in DWTP 2, whereas hypochlorite or chlorine gas was applied in other plants. ClO_2_ is known to produce less regulated DBPs and TOX than free chlorine,^6^ and this appears to be also true for the sulfonated DBPs found in this study.

BrANSA was relatively higher in DWTP 1, possibly related to a higher bromide concentration in its raw water. As the bromide concentration was below the LOQ (80 μg/L) in all samples in this study (Table S1), this remains open. DWTP 6 appears to have higher Cl_2_ANSA and BrClANSA than other plants. This may be related to the slightly higher chlorine dose compared to other plants (0.5–1 vs. 0.2–0.3 mg/L as Cl_2_) or the different quality of organic matter in the source water as here surface water was used.

DWTPs 3, 4, and 5 provide drinking water to the same city. The DBP mix in the finished water of DWTP may change in the distribution system due to dilution, hydrolysis, or enhanced contact time with a disinfectant.^40^ Therefore, additional samples were collected from two entry points to a drinking water distribution network and two drinking water storage reservoirs of this city (Figure S26). At these locations much closer to the consumer taps, total HANSAs were up to 2-fold higher (30–50 ng/L) than those produced at DWTPs. This outlines that HANSAs formation continues upon longer chlorine exposure.

Additionally, the booster chlorination (to maintain the residual chlorine in distribution system) can also increase the concentration of sulfonated DBPs. As an example, four different tap water samples were collected from different areas of one city: those taken from the area with the application of booster chlorination contained up to 50 and 130 ng/L HANSAs and HAcAlSAs, respectively, much higher than those without booster chlorination (<5 ng/L, Figure S27).

#### Tap Water

Figure 1b shows the novel sulfonated DBPs in tap water samples collected from 8 cities across Europe with different source waters (e.g., river bank filtration, groundwater, surface water) and treatment trains. At least one type of these DBPs (i.e., ClANSA) was present in all samples. The total HANSAs was varied from 1 ng/L to 50 ng/L, whereas HAcAlSAs were either undetectable (Stockholm, Uppsala) or can reach 800 ng/L (Prague). Particularly, the relative abundance of BrANSA was much higher in tap water of most cities compared to the samples taken from DWTPs in Figure 1a. This is possibly related to the higher bromine incorporation into DBPs with longer exposure time in distribution networks. However, it should be noted that the concentration of BrANSA here was estimated based on the response factor of its chlorinated analogue (^13^C_2_-^15^N-ClANSA). BrANSA and ClANSA may have different ionization efficiencies in the ESI source depending on their halogen type or show different recovery rates during enrichment. The SFC-QTOF signal sensitivity of ClMSA vs BrMSA and Cl_2_MSA vs Br_2_MSA was compared. In both cases, the sensitivity difference (slope of the linear curve of concentration vs. signal intensity) did not exceed the range of 2–3. On this basis, it appears acceptable to use the calibration curves of the chlorinated sulfonated DBPs for a first assessment of the concentration of structurally related brominated sulfonated DBPs. Nevertheless, the occurrence of brominated DBPs is of great interest as they are generally considered much more toxic than their chlorinated analogues.^8^

#### Swimming Pools

The novel sulfonated DBPs were also detected from three chlorinated public swimming pools (Figure S28). Cl_2_ANSA predominated in all studied samples in the range of 390–850 ng/L, followed by Cl_2_AcAlSA (340–770 ng/L). The concentrations of brominated species were less than 15 ng/L or even undetectable (i.e., Br_2_ANSA and BrClAcAlSA). This might be due to the low bromide level in filling waters, data of which is not available. Still, the concentrations of Cl_2_ANSA and Cl_2_AcAlSA in swimming pools were much higher than most tap water samples in Figure 1b (factor of >10 for Cl_2_AcAlSA), revealing that not only tap water consumers but also swimmers are potentially being exposed to these newly identified sulfonated DBPs.

## 4 Environmental Implication

Together with previous studies on HMSAs, our study shows that the sulfonic acid derivatives of all regularly monitored DBPs (i.e., halomethanes, haloacetonitriles, haloacetamides, and haloacetaldehydes) are present in disinfected water. This study also shows that sulfur containing moieties in organic matter, especially the reduced sulfur group in amino acid cysteine, peptides, and proteins can act as precursors of this class of compounds. Therefore, if a drinking water source is impacted by wastewater discharge or algal bloom, enriched in biomolecules, then the formation yield of these DBPs may increase upon chlorination. Moreover, due to the extreme acidity and polarity at any pH, these small sulfonated DBPs are likely overlooked during generic TOX measurements based on activated carbon adsorption and might be excluded in previous studies reporting the formation potential of total DBPs from a certain water source or disinfection process. Given the fact that haloacetonitriles, haloacetamides, and haloacetaldehydes are more cytotoxic and genotoxic than the regulated THMs and HAAs, their sulfonic acid derivatives found in this study might also pose a health threat and would require future studies on toxicity assessment.

This study shows that alternative chromatographic separation methods (e.g., SFC) when combined with HRMS can narrow the analytical gaps in monitoring highly polar substances, including the identification of unknown polar DBPs. The synthesis of DBPs by lab-scale chlorination of a known precursor followed by a combination of NMR and HRMS analysis as done in this study provides a possibility for the identification and quantification of novel DBPs in the case of no analytical standards availability. Such an approach can be extended to the identification of unknown products produced by other transformation processes (e.g., biotransformation, ozonation) during environmental monitoring.

## Supporting Information Available

Additional information on water samples, extracted ion chromatograms, MS/MS spectra, and ^13^C{^1^H} NMR spectra of compounds, and additional data from lab-scale chlorination experiments (PDF)
