# Supplementary material for: Revisiting Disinfection Byproducts with Supercritical Fluid Chromatography-High Resolution-Mass Spectrometry: Identification of Novel Halogenated Sulfonic Acids in Disinfected Drinking Water
Source: Environ Sci Technol. 2023 Feb 20;57(9):3527–37. doi: 10.1021/acs.est.2c05536 (PMC9996826; doi:10.1021/acs.est.2c05536)
Supplement: Supplementary file 1 — es2c05536_si_001.pdf [file es2c05536_si_001.pdf]

Supporting information for

Revisiting disinfection byproducts with supercritical fluid chromatography-high resolution-mass spectrometry: Identification of novel halogenated sulfonic acids in disinfected drinking water

Maolida Nihemaiti,[a,] * Maik Icker,[b] Bettina Seiwert,[a] Thorsten Reemtsma [a, c]

[a] Helmholtz Centre for Environmental Research - UFZ, Department of Analytical Chemistry, Permoserstrasse 15, 04318 Leipzig, Germany

[b] University of Leipzig, Institute of Organic Chemistry, Johannisallee 29, 04103 Leipzig, Germany

[c] University of Leipzig, Institute of Analytical Chemistry, Linnéstrasse 3, 04103 Leipzig, Germany

* Corresponding author: maolida.nihemaiti@ufz.de

Number of pages: 39

Number of tables: 3

Number of texts: 2

Number of figures: 28

Table S1. Total organic carbon (TOC) and bromide (Br^-) concentrations of drinking water treatment plant (DWTP) samples [a]

DWTP 1		DWTP 2	
Sampling date	TOC, Br^- (mg/L)	Sampling date	TOC, Br^- (mg/L)
22.03.2021	1.56, <0.08 (before Cl_2)<br>1.58, <0.08 (after Cl_2)	14.01.2021	3.28, 0.08 (before ClO_2)<br>2.82, <0.08 (after ClO_2)
06.04.2021	1.48, < 0.08 (before Cl_2)<br>1.39, <0.08 (after Cl_2)	28.01.2021	2.26, <0.08 (before ClO_2)<br>2.25, <0.08 (after ClO_2)
22.04.2021	1.51, <0.08 (before Cl_2)<br>1.85, <0.08 (after Cl_2)	11.02.2021	2.53, 0.08 (before ClO_2)<br>2.69, <0.08 (after ClO_2)
03.05.2021	1.54, <0.08 (before Cl_2)<br>1.54, <0.08 (after Cl_2)	25.02.2021	2.03, <0.08 (before ClO_2)<br>2.78, <0.08 (after ClO_2)
17.05.2021	1.63, <0.08 (before Cl_2)<br>1.63, <0.08 (after Cl_2)	11.03.2021	2.22, 0.08 (before ClO_2)<br>2.36, <0.08 (after ClO_2)

	DWTP 3	DWTP 4	DWTP 5
Sampling date		TOC, Br^- (mg/L)	
27.06.2021	0.80, <0.08 (before hypochlorite)<br>0.71, <0.08 (after hypochlorite)	0.94, <0.08 (before Cl_2)<br>0.95, <0.08 (after Cl_2)	1.01, <0.08 (before Cl_2)<br>1.18, <0.08 (after Cl_2)
29.09.2021	0.84, <0.08 (before hypochlorite)<br>0.81, <0.08 (after hypochlorite)	0.98, <0.08 (before Cl_2)<br>0.99, <0.08 (after Cl_2)	1.18, <0.08 (before Cl_2)<br>1.12, <0.08 (after Cl_2)
05.11.2021	1.06, <0.08 (before hypochlorite)<br>1.22, <0.08 (after hypochlorite)	1.18, <0.08 (before Cl_2)<br>1.22, <0.08 (after Cl_2)	1.35, <0.08 (before Cl_2)<br>1.41, <0.08 (after Cl_2)

DWTP 6 (29.06.2021), TOC (mg/L)			
catchment	after coagulation	after filtration	after Cl_2
1.16	0.90	0.83	0.94

[a] TOC is measured as NPOC (non purgeable organic carbon) using DIMATOC® 2100 from Dimatec Analysentechnik GmbH (Essen, Germany). Br^- is analysed using Dionex™ ICS-6000 Ion Chromatography (Thermo Scientific). The method limit of quantification is 80 µg/L.

Table S2. The ^{1}H, ^{13}C and ^{15}N chemical shifts of compounds identified by NMR analysis and quantified by qNMR

**Compound**	**^{13}C chemical shift* / ppm**	**^{1}H chemical shifts* / ppm**	**^{15}N chemical shift* / ppm**
urea (int. standard for quantification)	161.73 (s)	not visible due to fast exchange of NH_2 with D_2O	not determined in this mixture
$^{13}C_2$-^{15}N-ClANSA	116.41 (m, -**C**N), 53.98 (dd, *J* = 67.8, 3.7 Hz, -**C**H(Cl)-SO_3H)	3.69 (m, -C**H**(Cl)-SO_3H), SO_3H not visible due to fast exchange with D_2O	-132.4
$^{13}C_2$-^{15}N-Cl_2ANSA	116.41 (m, -**C**N), 74.66 (dd, *J* = 75.7, 4.0 Hz, -**C(**Cl_2)-SO_3H)	SO_3H not visible due to fast exchange with D_2O	-132.4
$^{13}C_2$-Cl_2AcAlSA	186.21 (d, *J* = 38.5 Hz, -**C**HO); 92.93 (d, *J* = 38.5 Hz, -**C(**Cl_2)-SO_3H	9.42 ((dd, *J* =203.0, 38.0 Hz, -C**H**O), SO_3H not visible due to fast exchange with D_2O	./.

* The ^{1}H NMR signal of CD_3CN was referenced to 1.94 ppm. ^{13}C and ^{15}N chemical shift scales have been referenced by calculating a reference signal with a Ξ value of 25.145020 and 10.13767 ($MeNO_2$ = 0 ppm) respectively.

Table S3. Details on qNMR data. The concentrations of compounds were calculated based on their relative peak area to internal standard (urea).

Compound	^{13}C peak position [ppm]	Integral	Mass [µg]	Mass conc. [µg/L]	Molar conc. [µmol/L]	Molar weight [g/mol]	Volume [mL]
urea (int. quantification standard)	161	1.0000	25.84	112348	1840	61.048	0.23
$^{13}C_2$-^{15}N-ClANSA	52.9; 115	0.7698	19.89	86485	549	157.537	
$^{13}C_2$-^{15}N-Cl_2ANSA	185; 91.3	0.2415	6.24	27129	139	194.979	
$^{13}C_2$-Cl_2AcAlSA	73.6	0.6712	17.34	75408	391	192.972	

Text S1. Structure elucidation by NMR and assignment of NMR signals (description of Figure S23)

The usage of fully ^{13}C and ^{15}N cysteine tremendously simplifies the structure elucidation (and NMR quantification) of the transformation products not only due to the increased sensitivity of NMR spectroscopy methods. It also provides better insights into the carbon connectivity by C,C- and C,N-spin-spin couplings, which would not be visible with ^{13}C and ^{15}N at natural abundance.

Compounds $^{13}C_2$-^{15}N-ClANSA, $^{13}C_2$-^{15}N-Cl_2ANSA and $^{13}C_2$-Cl_2AcAlSA were identified with the help of their respective ^{13}C chemical shifts combined with 2D NMR correlation spectra (COSY, HSQC and HMBC). Heteronuclear 2D NMR experiments (HSQC and HMBC) allow for the entanglement of particular 13C signals with their 1H chemical shifts which provide characteristic chemical information. 15N chemical shifts have been acquired with the help of 1H,15N-HMQC NMR spectra.

Cyano groups give rise to typical 13C and 15N signals at 116 ppm and -132 ppm, respectively. The13C signals of aldehyde groups show chemical shifts in the range of 185 to 200 ppm. The difference in chemical shift between mono- and dichloro species is substantial, which enables a general group assignment within the 13C spectrum as the carbon spins of the dichloro species experience a much stronger deshielding effect (shift range Cl2-C: 70 to 98 ppm; shift range Cl-C: 53 to 58 ppm).

With the help of a 13C,13C-COSY experiment and the 13C spin-spin coupling constants (JCC) the attached carbons can be assigned which in turn provide details on their chemical environment due to their chemical shift value. The latter allows the identification of the substituents or H atoms being attached to them. With the help of an HSQC experiment it is straightforward to clarify whether or not the C atom also bears H atoms. Having identified a specific H atom the HMBC provides information on further C atoms in the constitution of the molecule, which are two and three bonds away from a particular H atom. Furthermore, correlation signals between 15N and 1H provide further insights on attached nitrogen containing groups to certain carbon atoms.

Text S2. Quantification using qNMR

Quantification by NMR spectroscopy is generally possible (usage of qNMR is constantly growing) and can be conducted by different means, e.g. by addition of an internal standard with known concentration. The latter was applied in current study. It is uncommon to choose 13C as a nuclear spin for quantification. However, being aware of certain properties like long 13C relaxation times and applying a proper NMR experiment (pulse sequence), it is absolutely safe to also employ 13C NMR spectroscopy for quantification.

The $^{13}C_3$-^{15}N labeled cysteine (1 mM) was chlorinated for 48 h by applying 5 mM of initial chlorine in 200 mL of phosphate buffer solution (10 mM, pH 7). The solution was then enriched by freeze-drying and re-constituted in acetonitrile-d3 and water-d2 (9:1, v:v). An aliquot (230 µL) of this extract (DBPs mixture) was analysed using NMR after being spiked with 100 µL of ^{13}C-urea (4.23 mM), an internal standard.

As ^{13}C spin relaxation times vary considerably throughout different species, this DBPs mixture was also spiked with the so-called relaxation agent, 2 mg of $Cr(acac)_3$. The number of scans and the repetition delay between each scan have been chosen accordingly to gain sufficient SNR (Figure S23).

As given in Table S3, the signal intensity (*i.e.* integrals) of the DBP ^{13}C peak was determined relative to internal standard which directly gives access to the amount of substance (in mol), as each ^{13}C NMR signal arises from a certain DBP: 549 µM, 391 µM, 139 µM for $^{13}C_2$-^{15}N-ClANSA, $^{13}C_2$-^{15}N-Cl_2ANSA, and $^{13}C_2$-Cl_2AcAlSA, respectively.

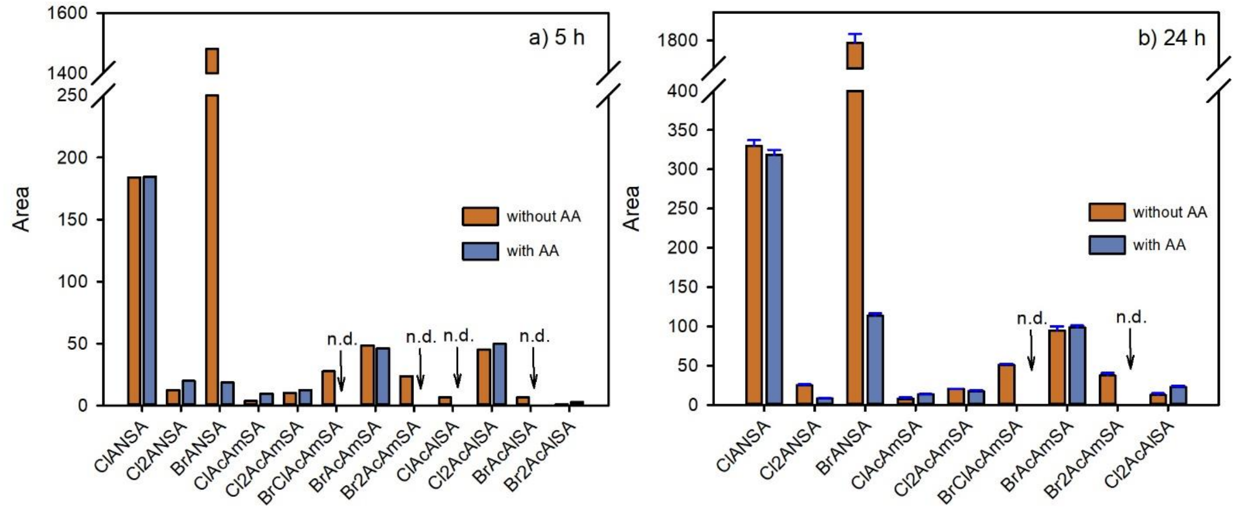

Figure S1. Effect of ascorbic acid (AA) on the stability of sulfonic acid DBPs identified in this study. Experiments were conducted using 40 mL aliquots of the disinfected water samples from DWTP 1 (samples collected on 22.04 and 22.03 were used in (a) and (b), respectively. No residual chlorine was detectable while conducing these experiments). The samples were spiked with 40 µL of AA solution (17 g/L in ultrapure water) and kept for 5 h (a) or 24 h (b) in the dark at room temperature, then analysed using SFC-QTOF following freeze-drying enrichment. Control samples were prepared following the same procedure but without spiking AA. Error bars in (b) represent the standard deviations of duplicate analysis. n.d.: non-detectable.

Results indicate that several DBPs (i.e., BrANSA, BrClAcAmSA, Br_2AcAmSA, ClAcAlSA, BrAcAlSA) were largely reduced or not detectable after being exposed to AA for 5 h and 24 h.

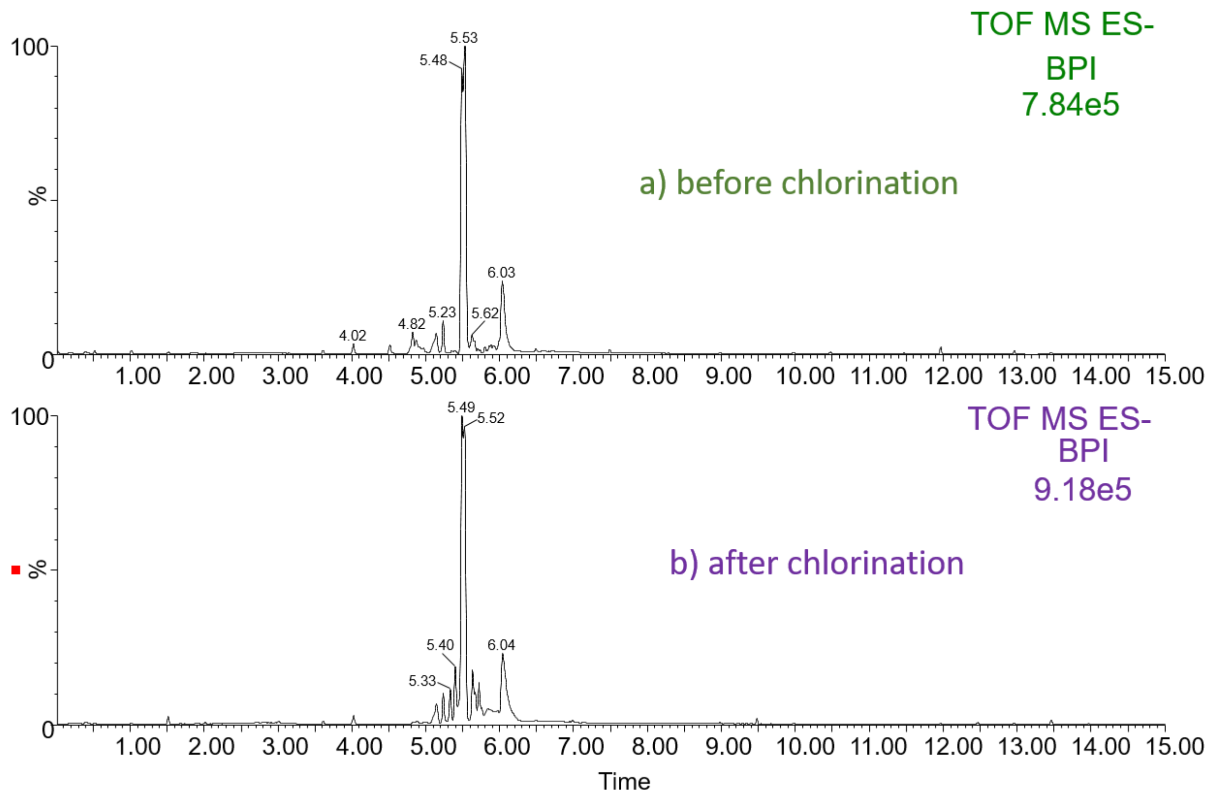

Figure S2. The base peak chromatogram of drinking water samples from DWTP 1 before (a) and after (b) chlorination. Samples were analysed using SFC-QTOF in negative ionization mode. The extracted ion chromatogram of each new DBP is shown in Figures S3-S17.

a)

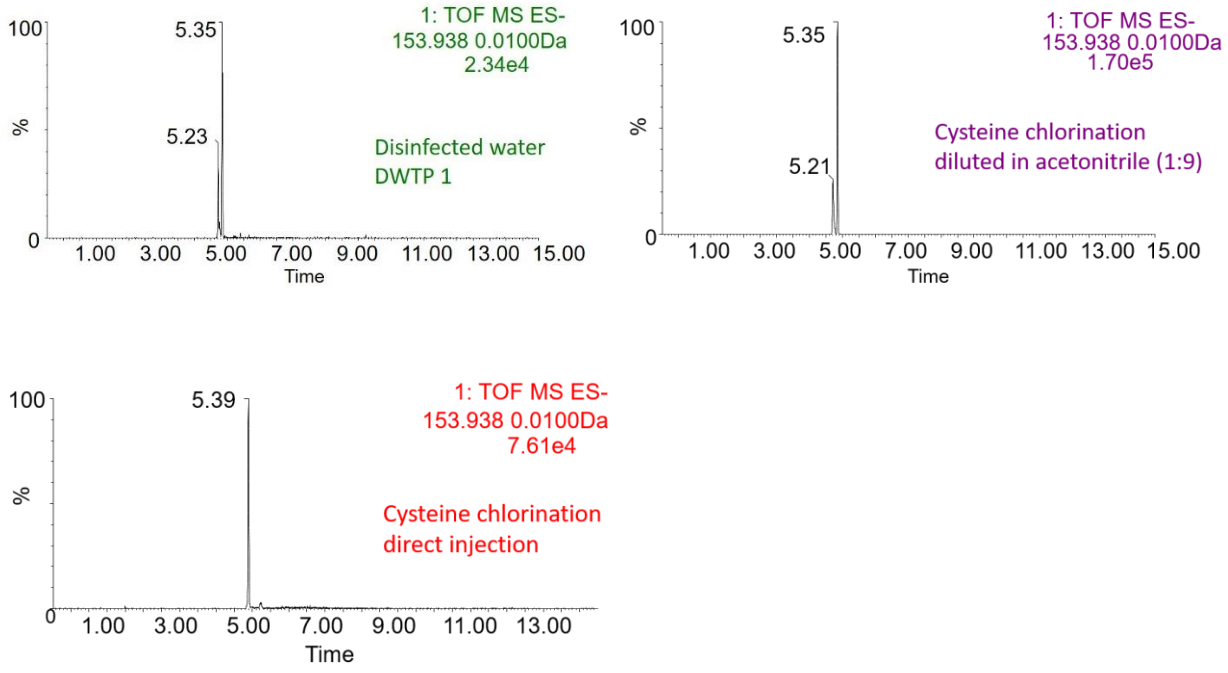

b)

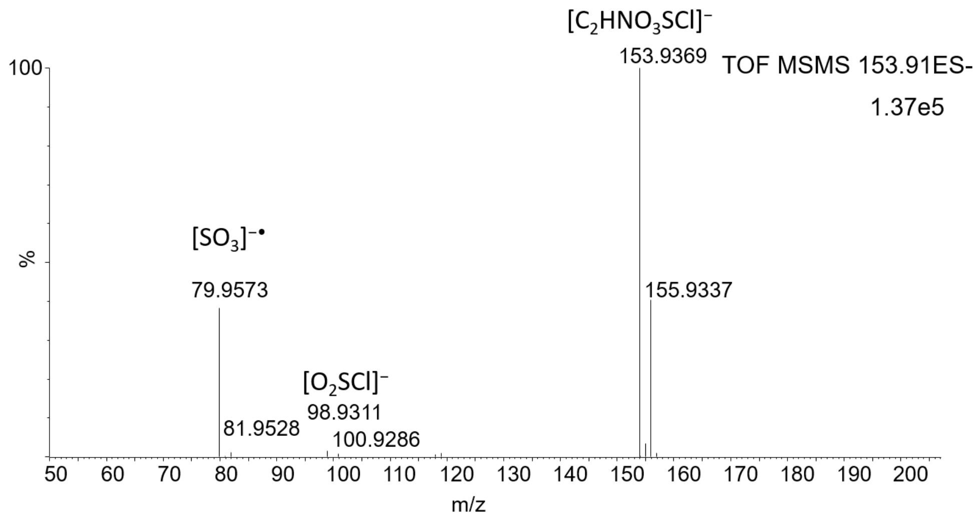

Figure S3. a) The extracted ion chromatogram of *m/z* 153.938 obtained during SFC-QTOF analysis of disinfected water from DWTP 1 and cysteine chlorination (diluted in acetonitrile or direct injection), and b) MS/MS spectrum of *m/z* 153.9369

a)

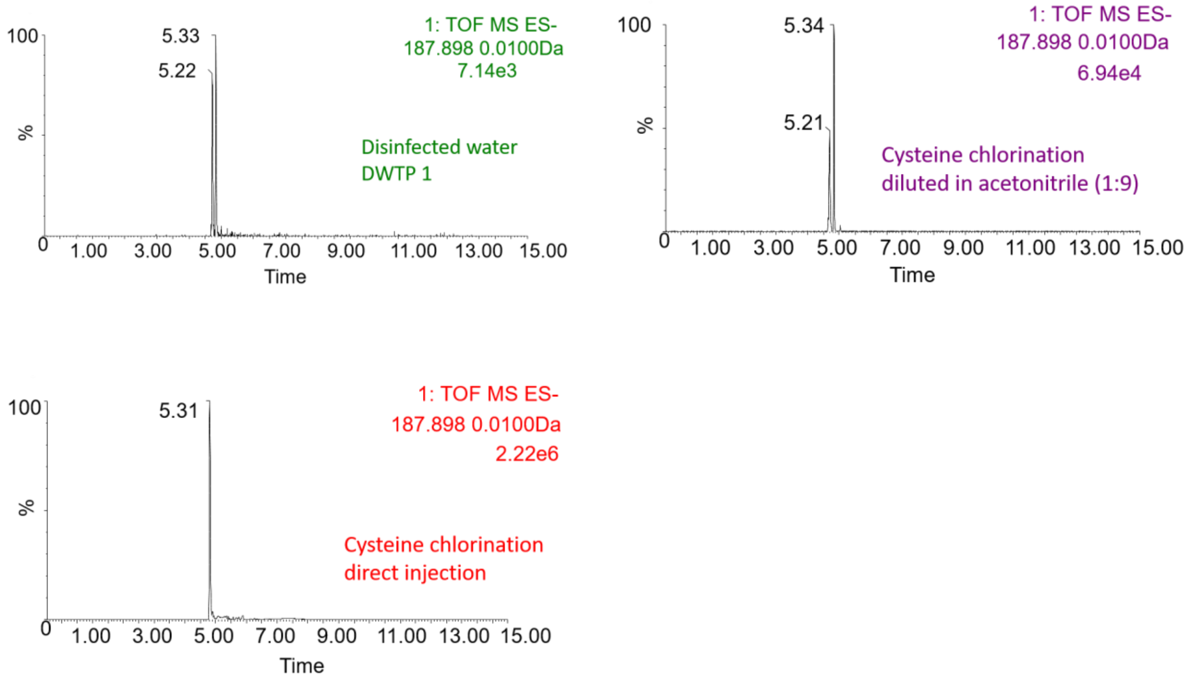

b)

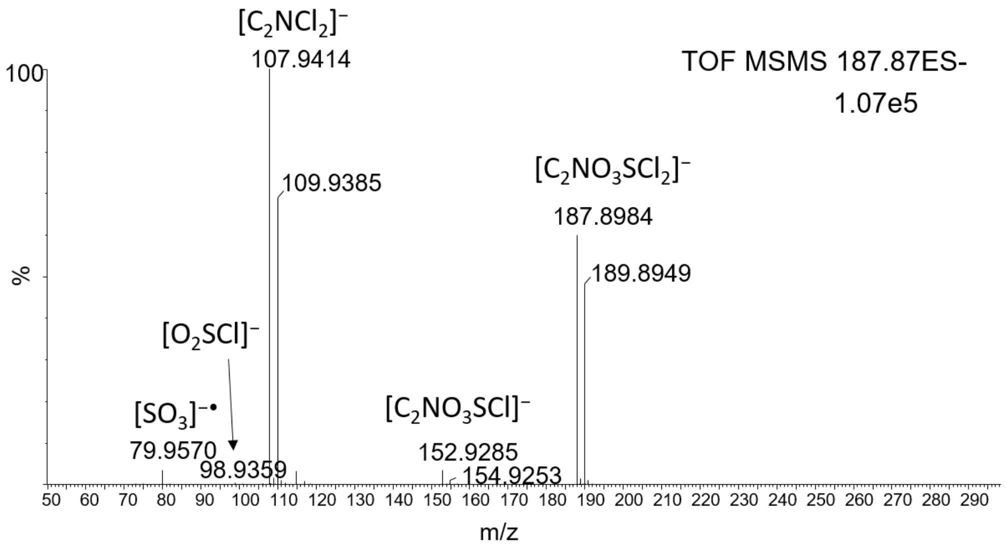

Figure S4. a) The extracted ion chromatogram of *m/z* 187.898 obtained during SFC-QTOF analysis of disinfected water from DWTP 1 and cysteine chlorination (diluted in acetonitrile or direct injection), and b) MS/MS spectrum of *m/z* 187.8984

a)

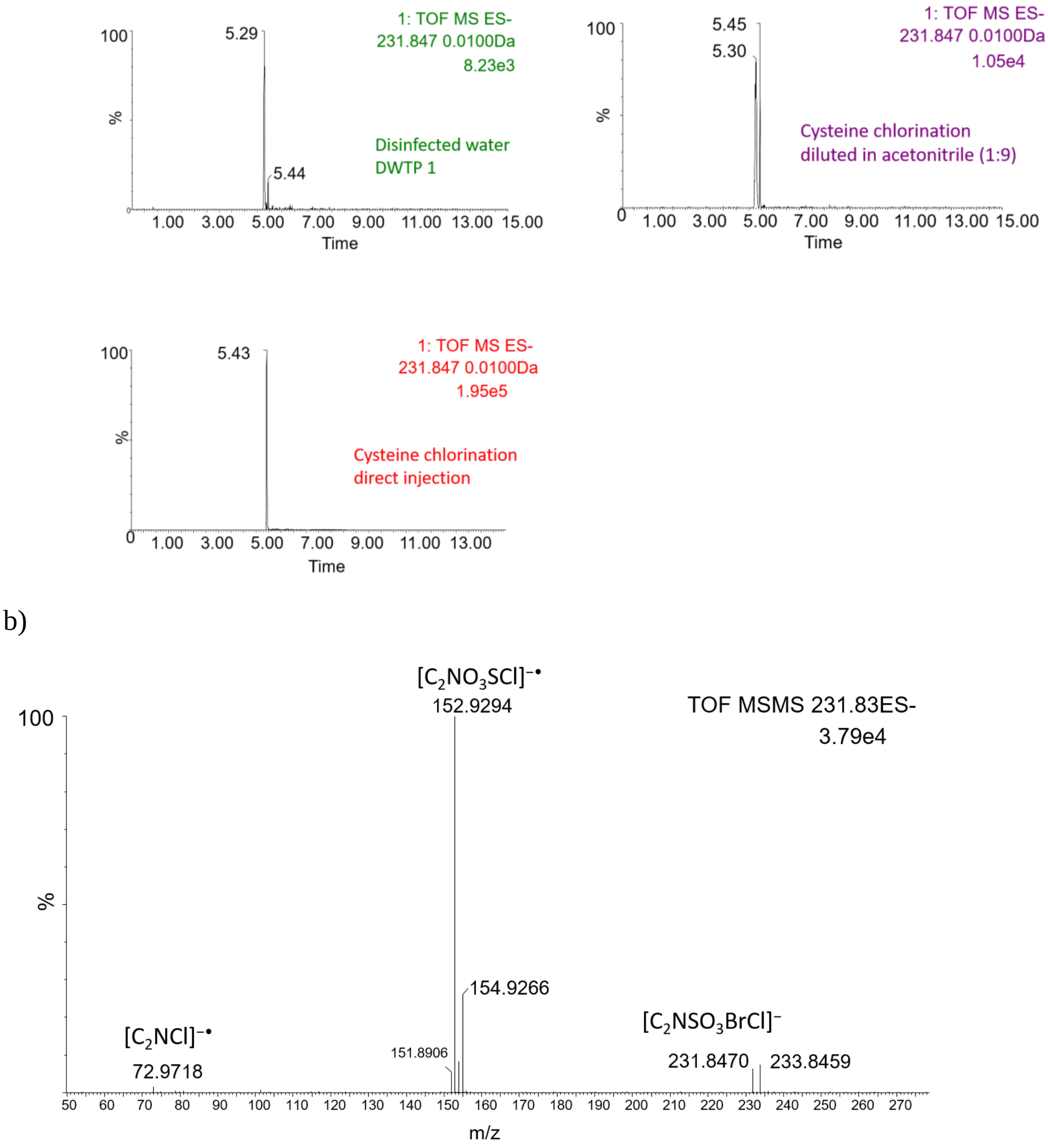

Figure S5. a) The extracted ion chromatogram of *m/z* 231.847 obtained during SFC-QTOF analysis of disinfected water from DWTP 1 and cysteine chlorination (diluted in acetonitrile or direct injection), and b) MS/MS spectrum of *m/z* 231.8470

a)

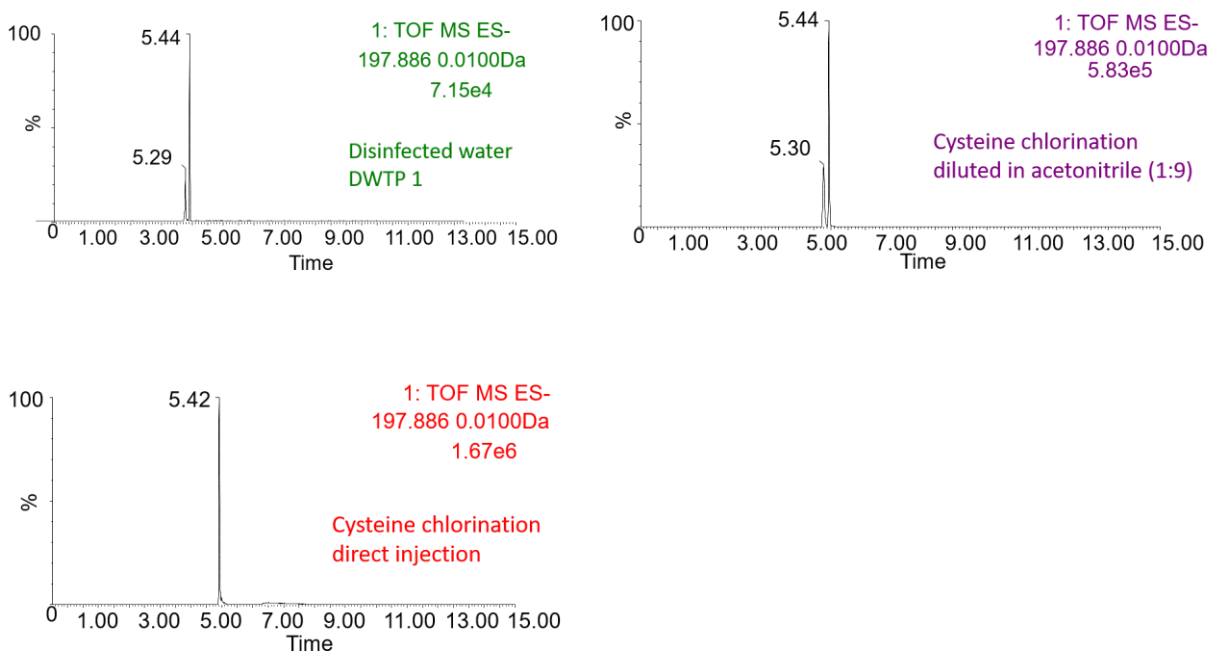

b)

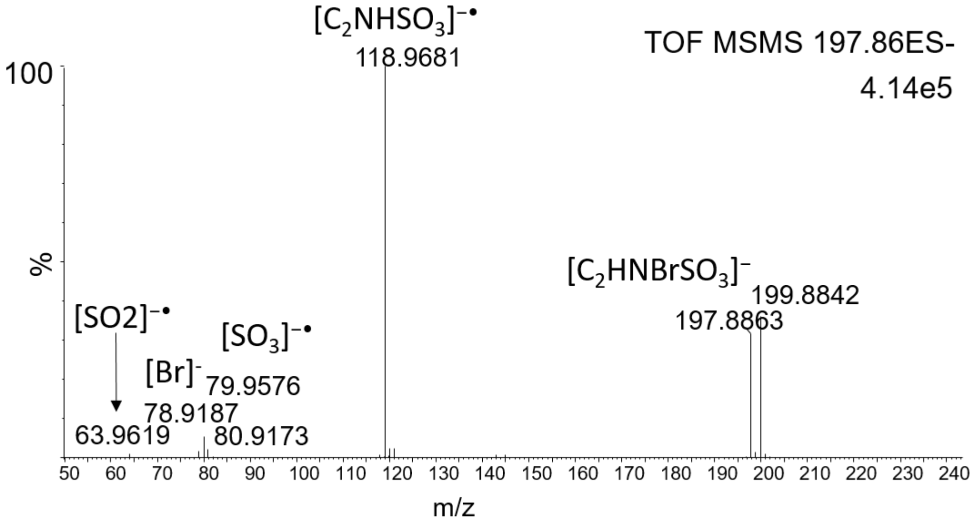

Figure S6. a) The extracted ion chromatogram of *m/z* 197.886 obtained during SFC-QTOF analysis of disinfected water from DWTP 1 and cysteine chlorination (diluted in acetonitrile or direct injection), and b) MS/MS spectrum of *m/z* 197.8863

a)

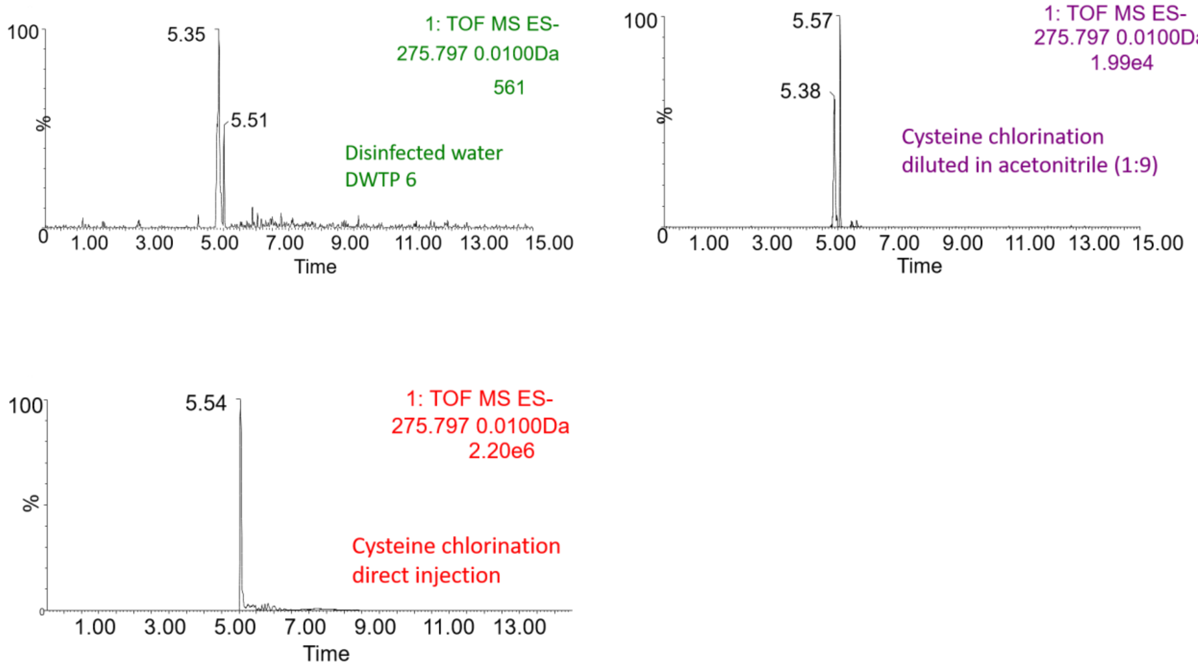

b)

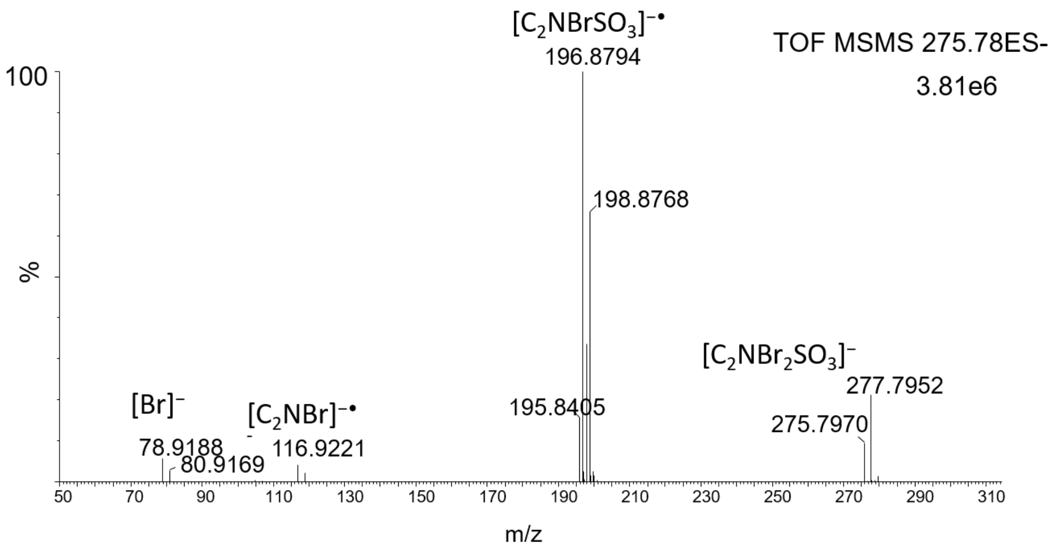

Figure S7. a) The extracted ion chromatogram of *m/z* 275.797 obtained during SFC-QTOF analysis of disinfected water from DWTP 6 and cysteine chlorination (diluted in acetonitrile or direct injection), and b) MS/MS spectrum of *m/z* 275.7970

a)

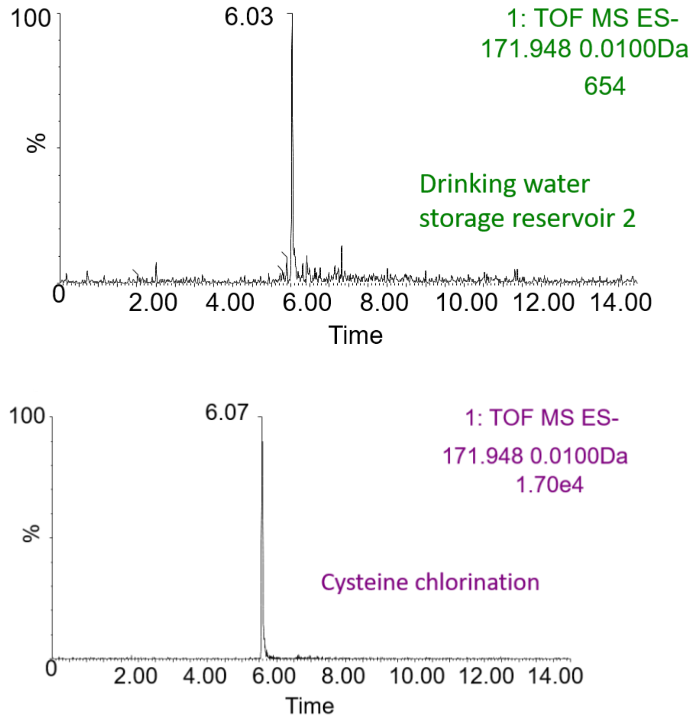

b)

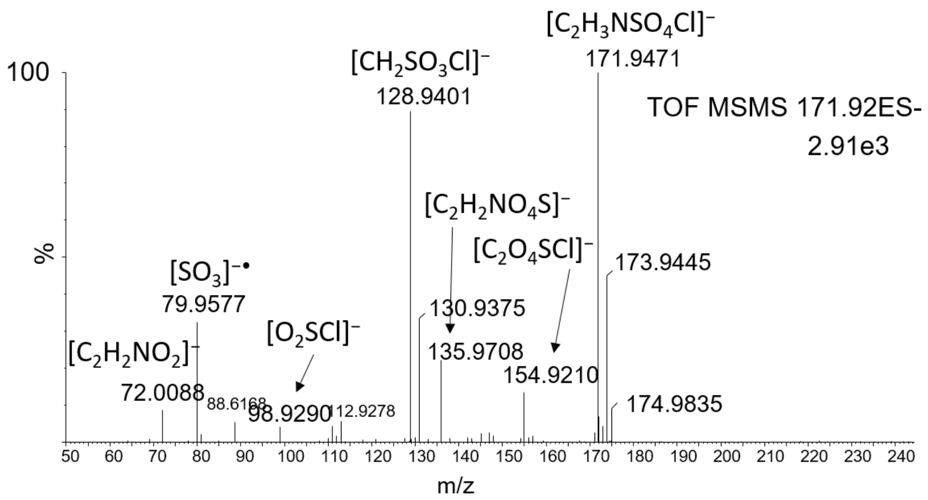

Figure S8. a) The extracted ion chromatogram of *m/z* 171.948 obtained during SFC-QTOF analysis of drinking water storage reservoir 2 and cysteine chlorination, and b) MS/MS spectrum of *m/z* 171.9471

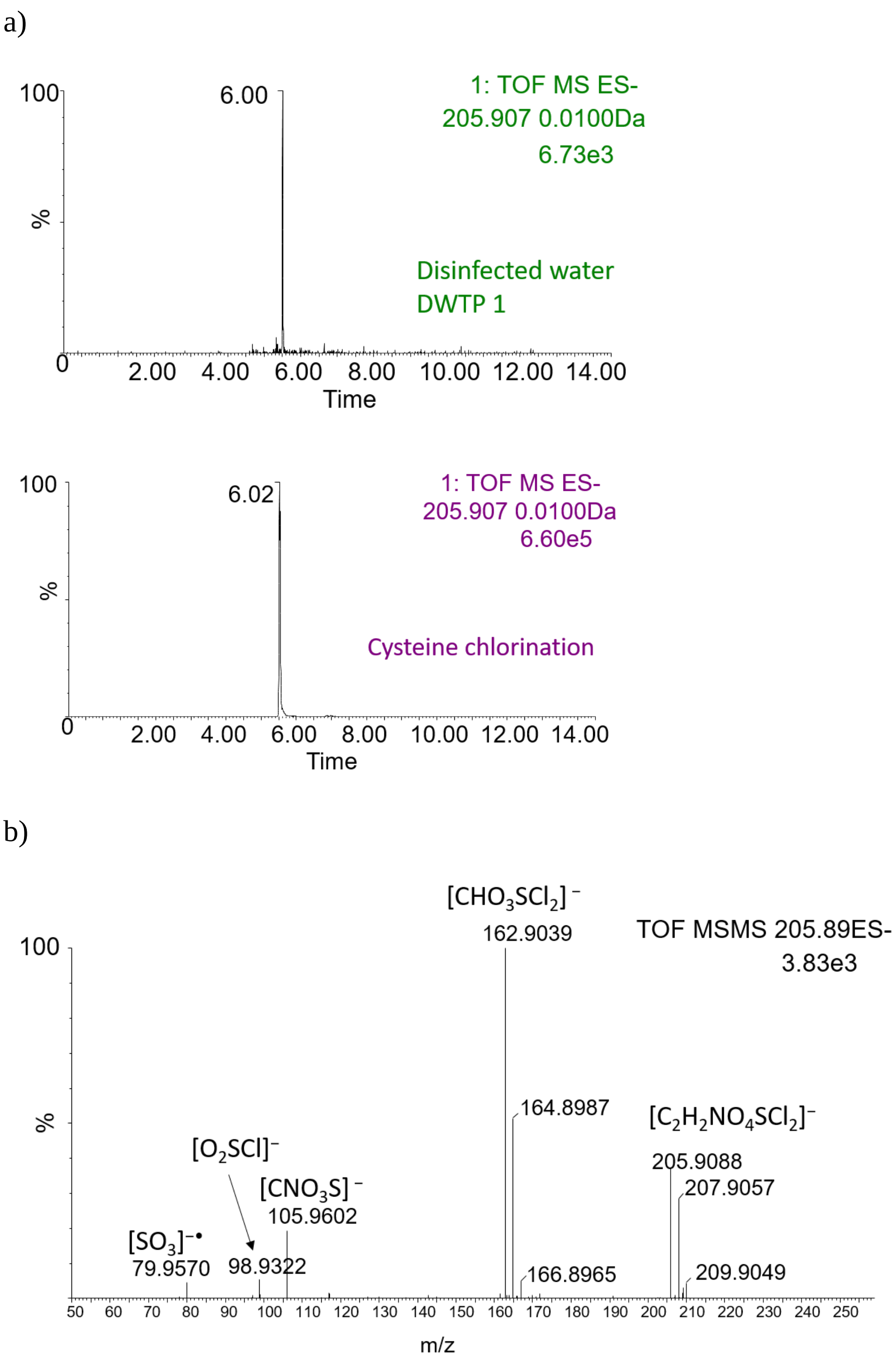

Figure S9. a) The extracted ion chromatogram of *m/z* 205.907 obtained during SFC-QTOF analysis of DWTP 1 and cysteine chlorination, and b) MS/MS spectrum of *m/z* 205.9088

a)

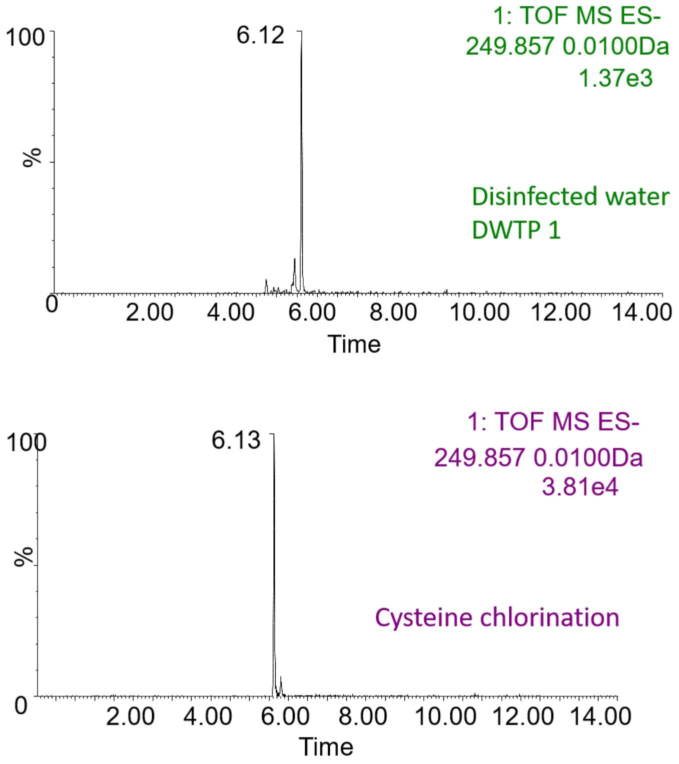

b)

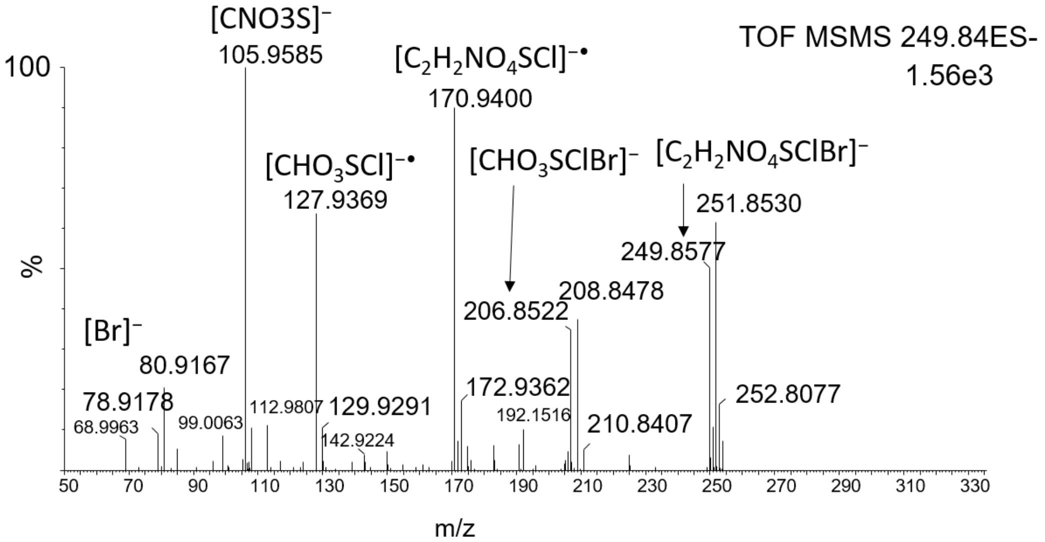

Figure S10. a) The extracted ion chromatogram of *m/z* 249.857 obtained during SFC-QTOF analysis of DWTP 1 and cysteine chlorination, and b) MS/MS spectrum of *m/z* 249.8577

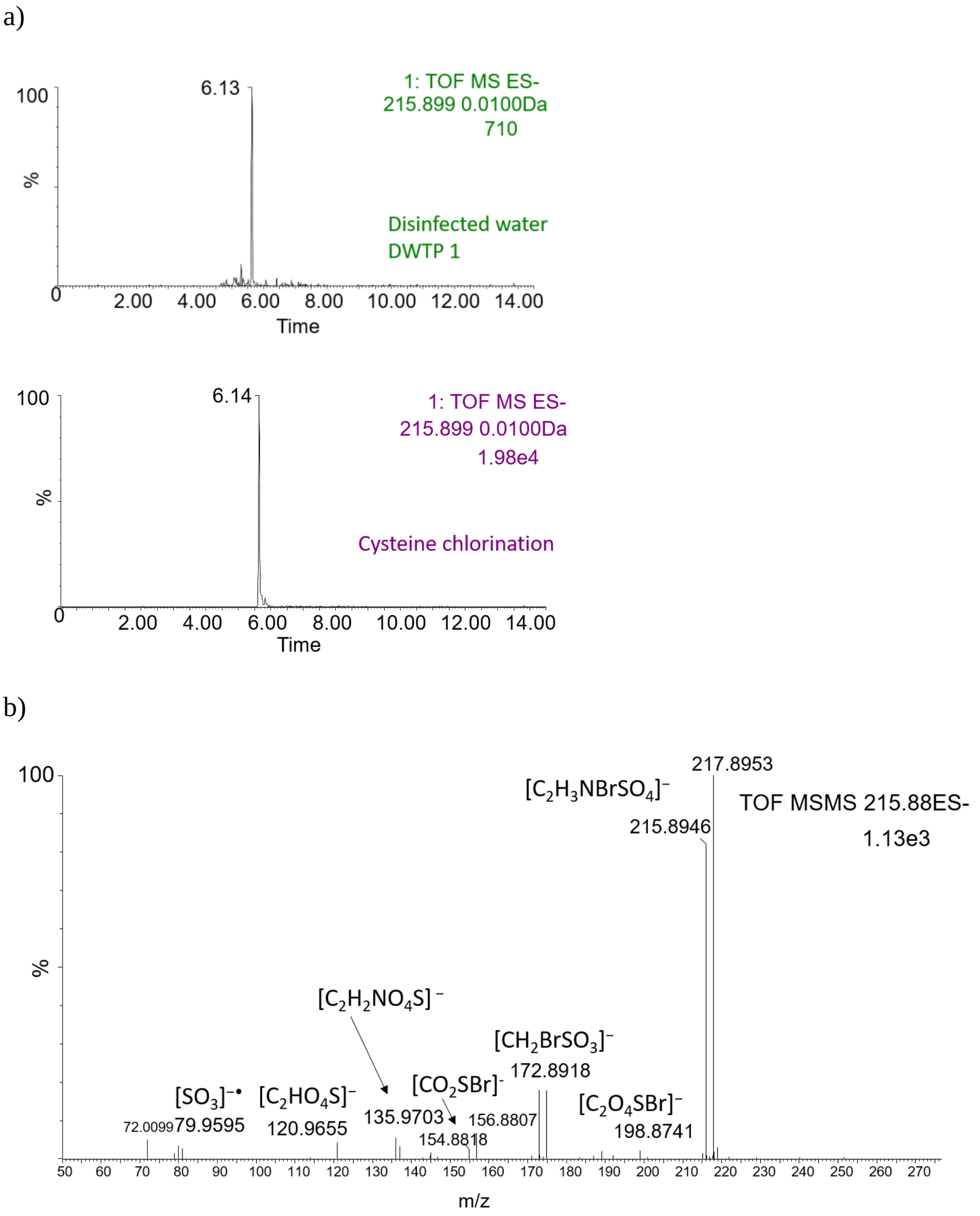

Figure S11. a) The extracted ion chromatogram of *m/z* 215.899 obtained during SFC-QTOF analysis of DWTP 1 and cysteine chlorination, and b) MS/MS spectrum of *m/z* 215.8946

a)

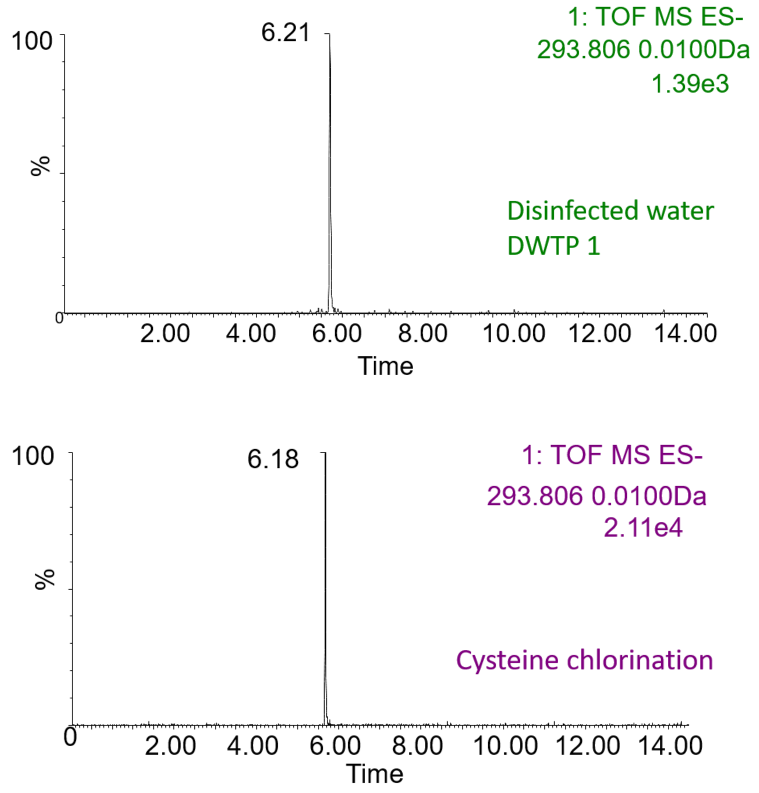

b)

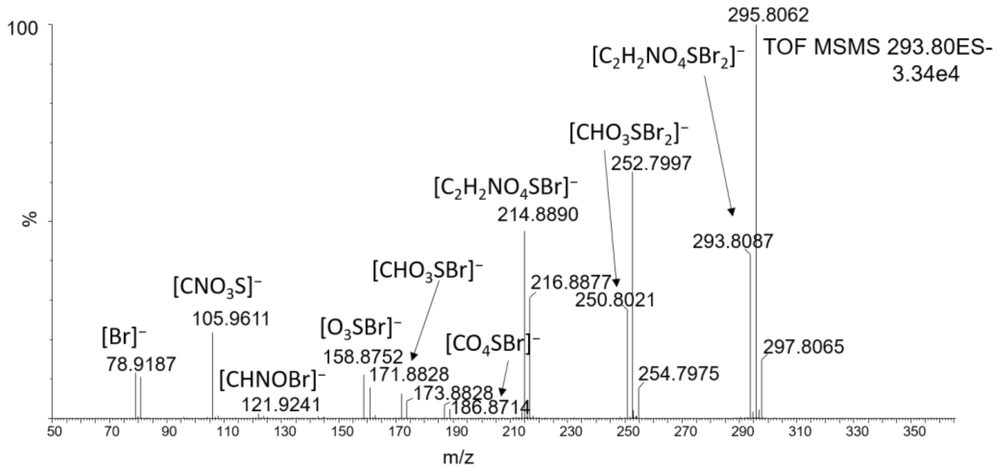

Figure S12. a) The extracted ion chromatogram of *m/z* 293.806 obtained during SFC-QTOF analysis of DWTP 1 and cysteine chlorination, and b) MS/MS spectrum of *m/z* 293.8087

a)

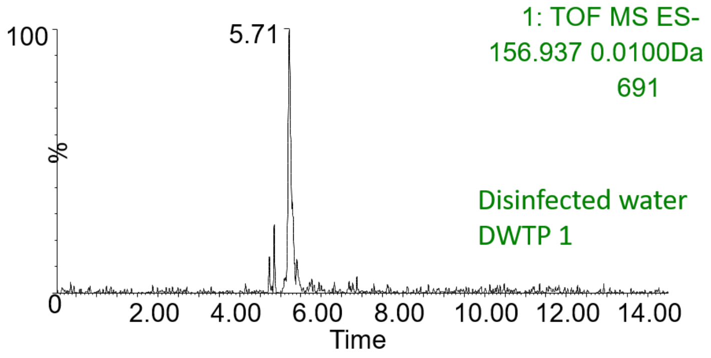

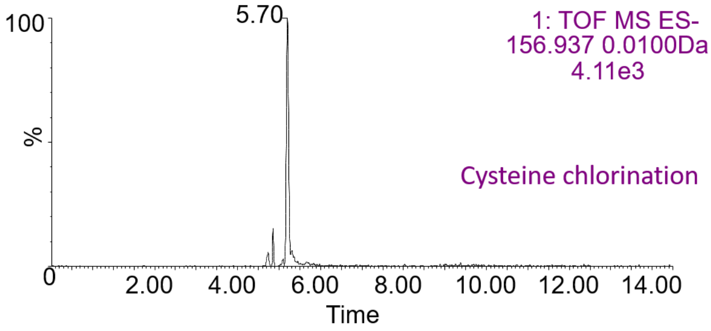

b)

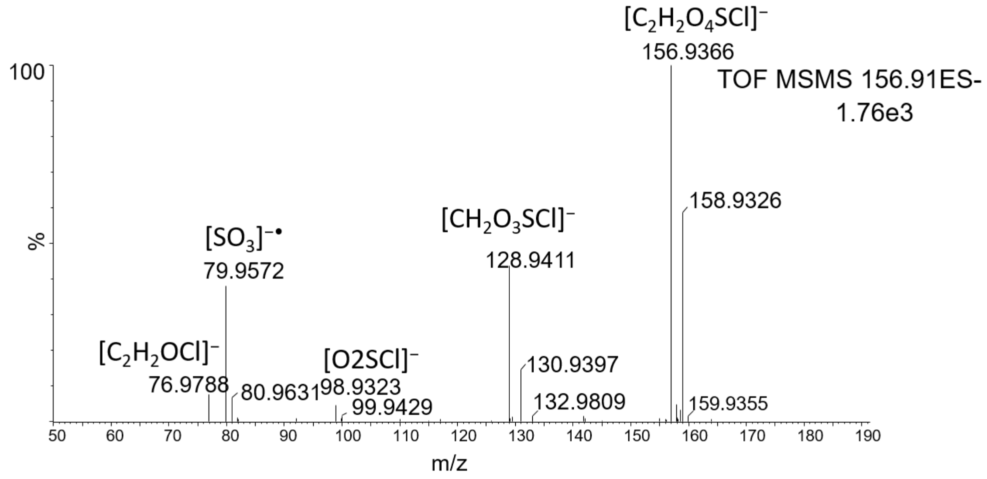

Figure S13. a) The extracted ion chromatogram of *m/z* 156.937 obtained during SFC-QTOF analysis of DWTP 1 and cysteine chlorination, and b) MS/MS spectrum of *m/z* 156.9366

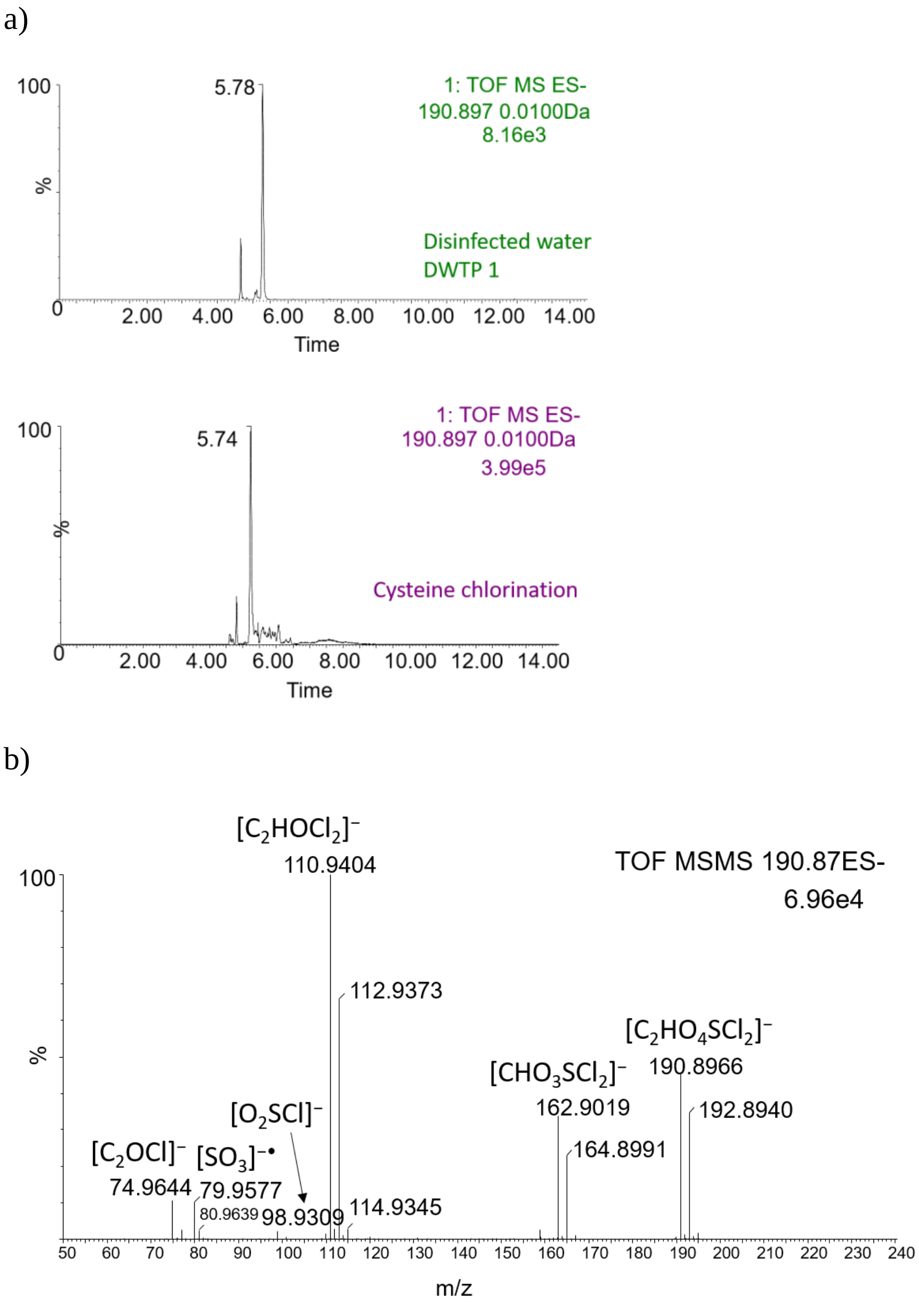

Figure S14. a) The extracted ion chromatogram of *m/z* 190.897 obtained during SFC-QTOF analysis of DWTP 1 and cysteine chlorination, and b) MS/MS spectrum of *m/z* 190.8966

a)

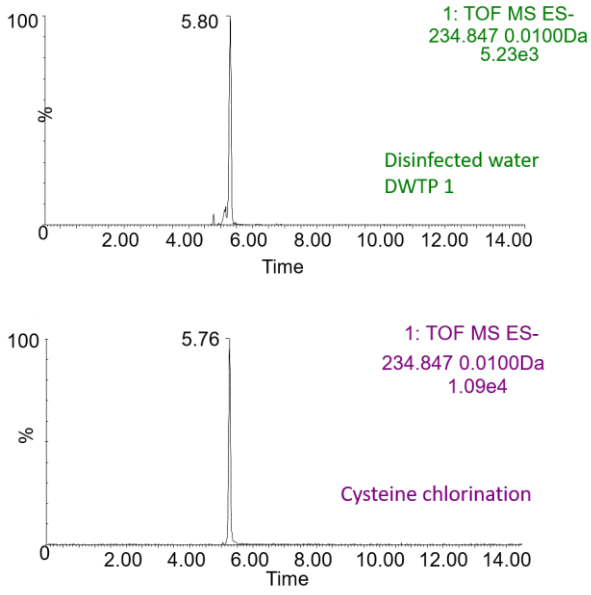

b)

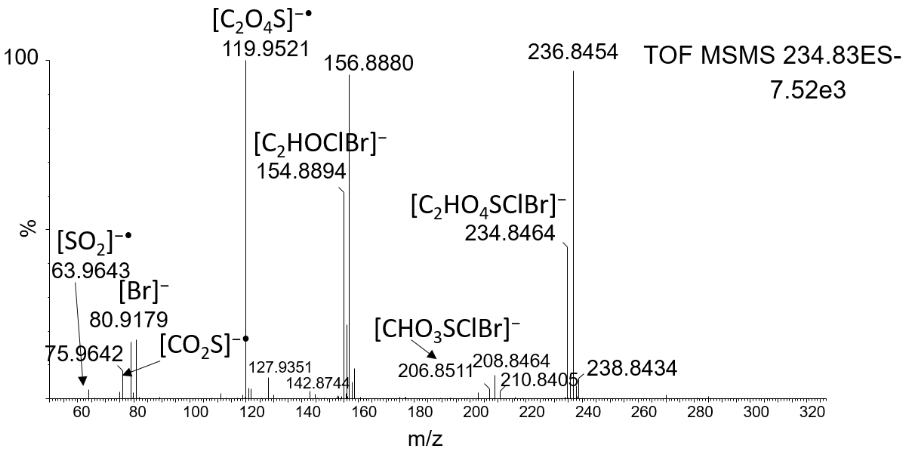

Figure S15. a) The extracted ion chromatogram of *m/z* 234.847 obtained during SFC-QTOF analysis of DWTP 1 and cysteine chlorination, and b) MS/MS spectrum of *m/z* 234.8464

a)

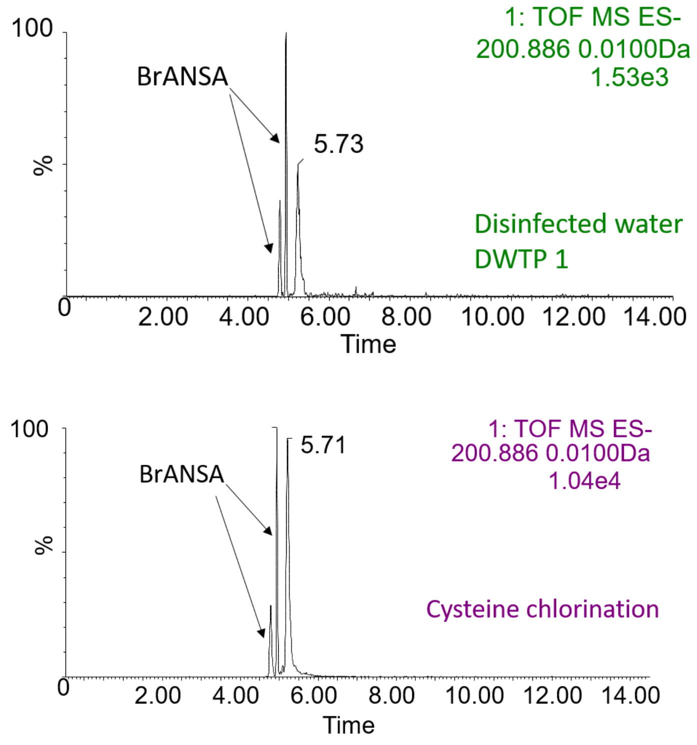

b)

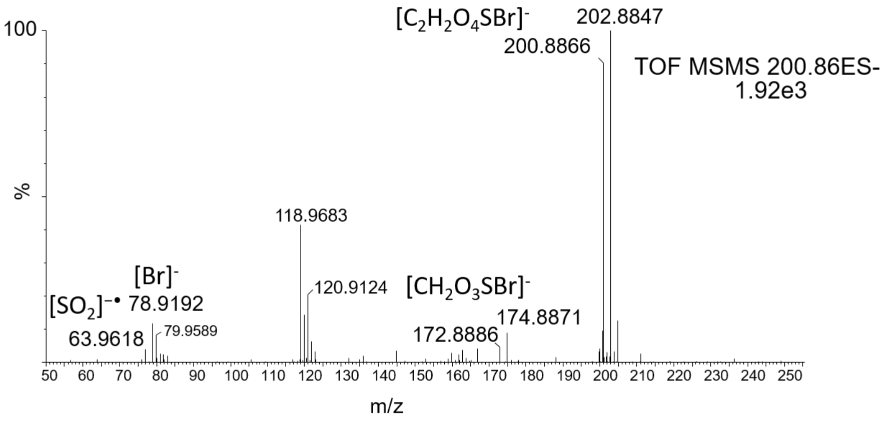

Figure S16. a) The extracted ion chromatogram of *m/z* 200.886 obtained during SFC-QTOF analysis of DWTP 1 and cysteine chlorination, and b) MS/MS spectrum of *m/z* 200.8866

a)

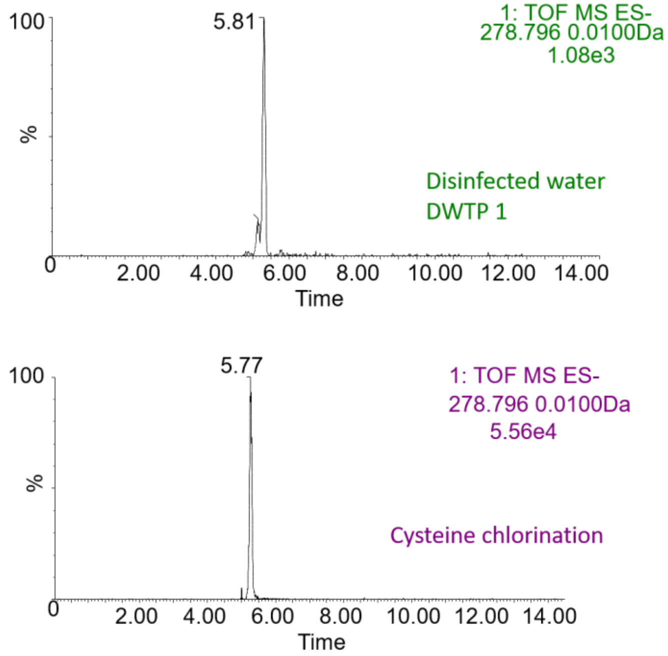

b)

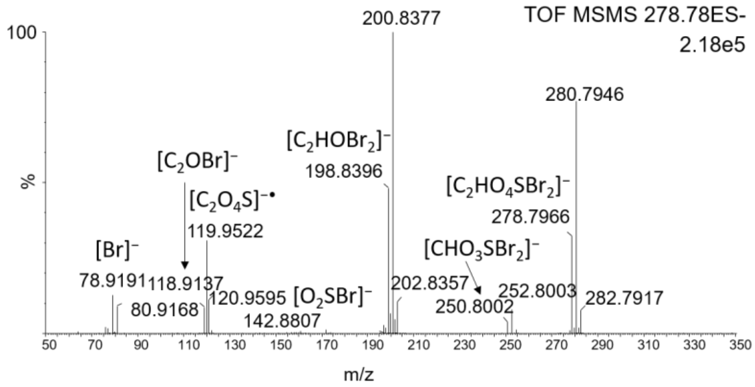

Figure S17. a) The extracted ion chromatogram of *m/z* 278.796 obtained during SFC-QTOF analysis of DWTP 1 and cysteine chlorination, and b) MS/MS spectrum of *m/z* 278.7966

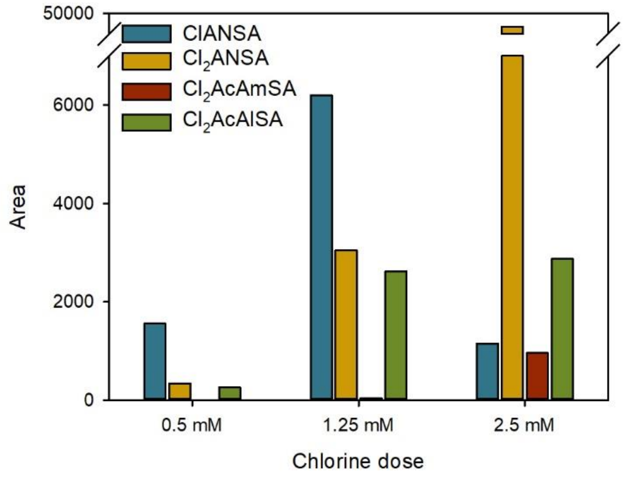

a)

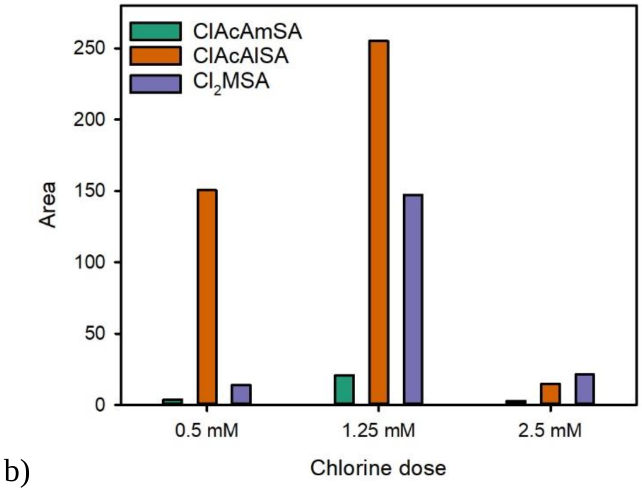

b)

Figure S18. Peak area of sulfonic acid DBPs (a and b) produced from the chlorination of cysteine (initial cysteine: 250 µM, initial chlorine doses: 0.5, 1.25, and 2.5 mM, reaction time: 5 h, 10 mM of phosphate buffer at pH 7). Samples were analysed by direct injection on SFC-QTOF without further enrichment. ClANSA- chloroacetonitrilesulfonic acid, Cl_2ANSA- dichloroacetonitrilesulfonic acid, ClAcAmSA- chloroacetamidesulfonic acid, Cl_2AcAmSA- dichloroacetamidesulfonic acid, ClAcAlSA- chloroacetaldehydesulfonic acid, Cl_2AcAlSA- dichloroacetaldehydesulfonic acid, Cl_2MSA- dichloromethanesulfonic acid.

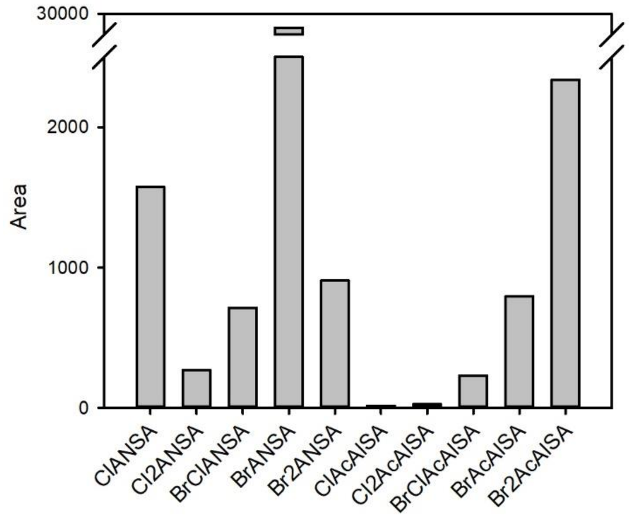

a)

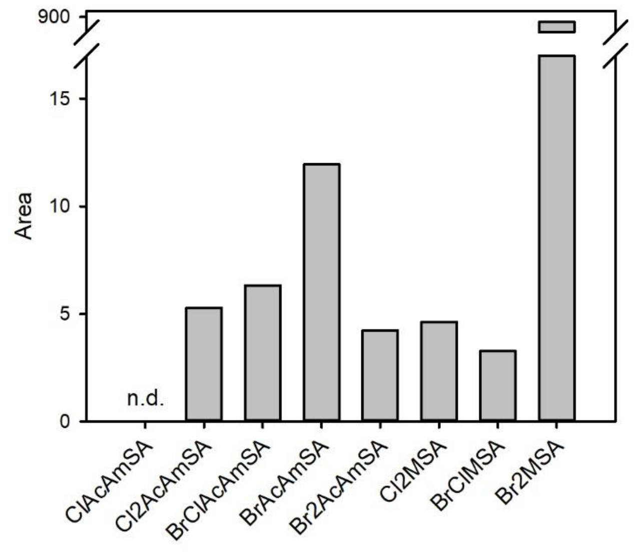

b)

Figure S19. Peak area of sulfonic acid DBPs (a and b) produced from the chlorination of cysteine in the presence of bromide ion (initial cysteine: 250 µM, initial chlorine dose: 1.25 mM, bromide: 500 µM, reaction time: 5 h, 10 mM of phosphate buffer at pH 7). n.d.: not detected. Sample was analysed by direct injection on SFC-QTOF without further enrichment.

a)

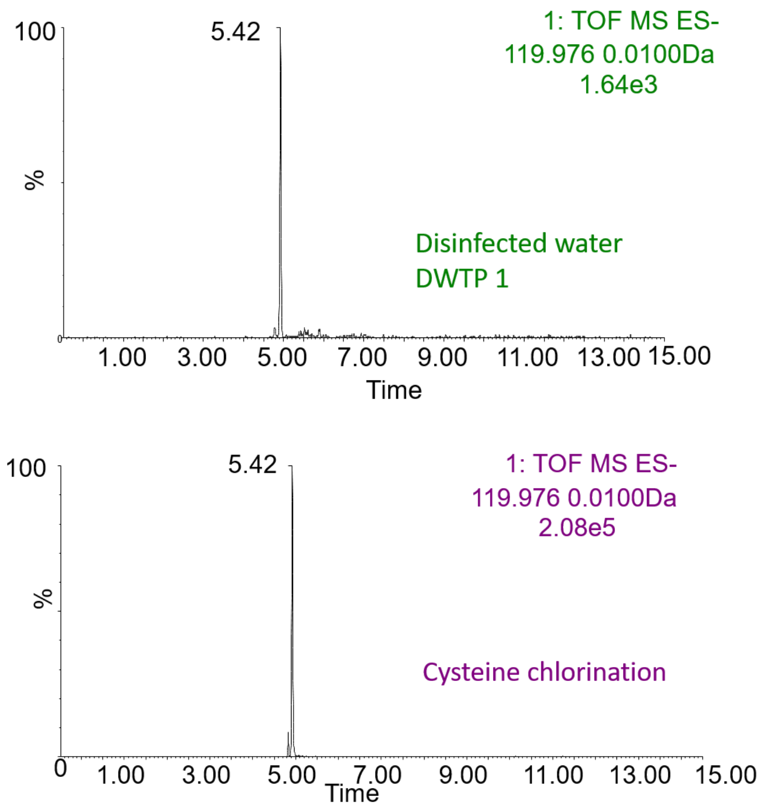

b)

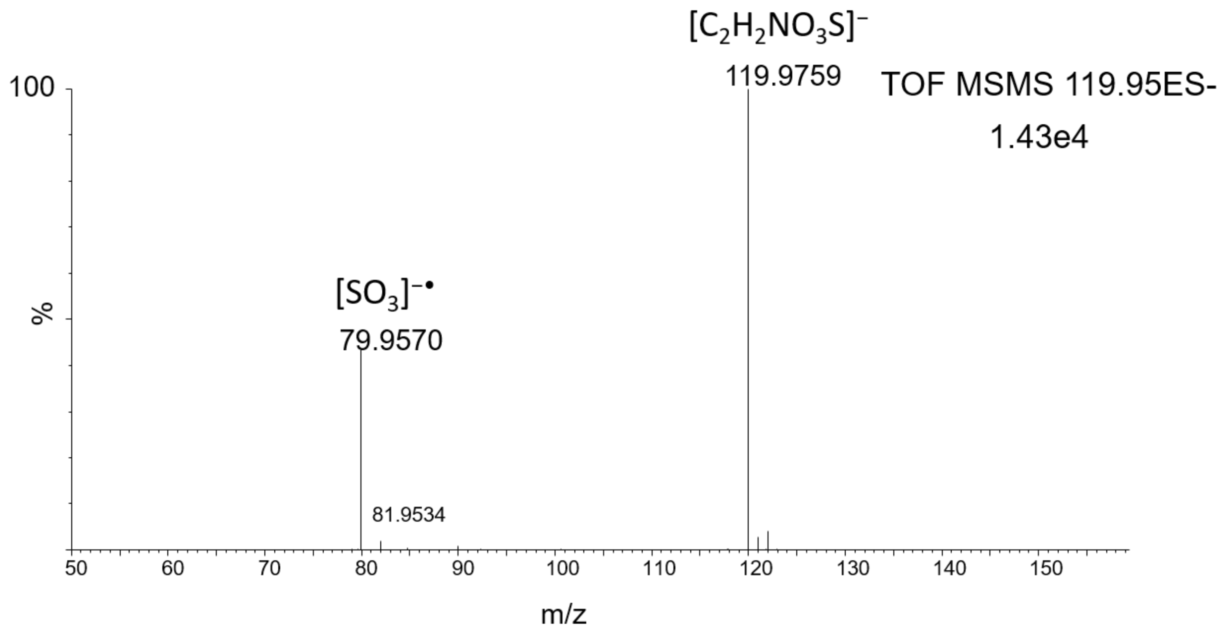

Figure S20. a) The extracted ion chromatogram of *m/z* 119.976 obtained during SFC-QTOF analysis of DWTP 1 and cysteine chlorination, and b) MS/MS spectrum of *m/z* 119.9759

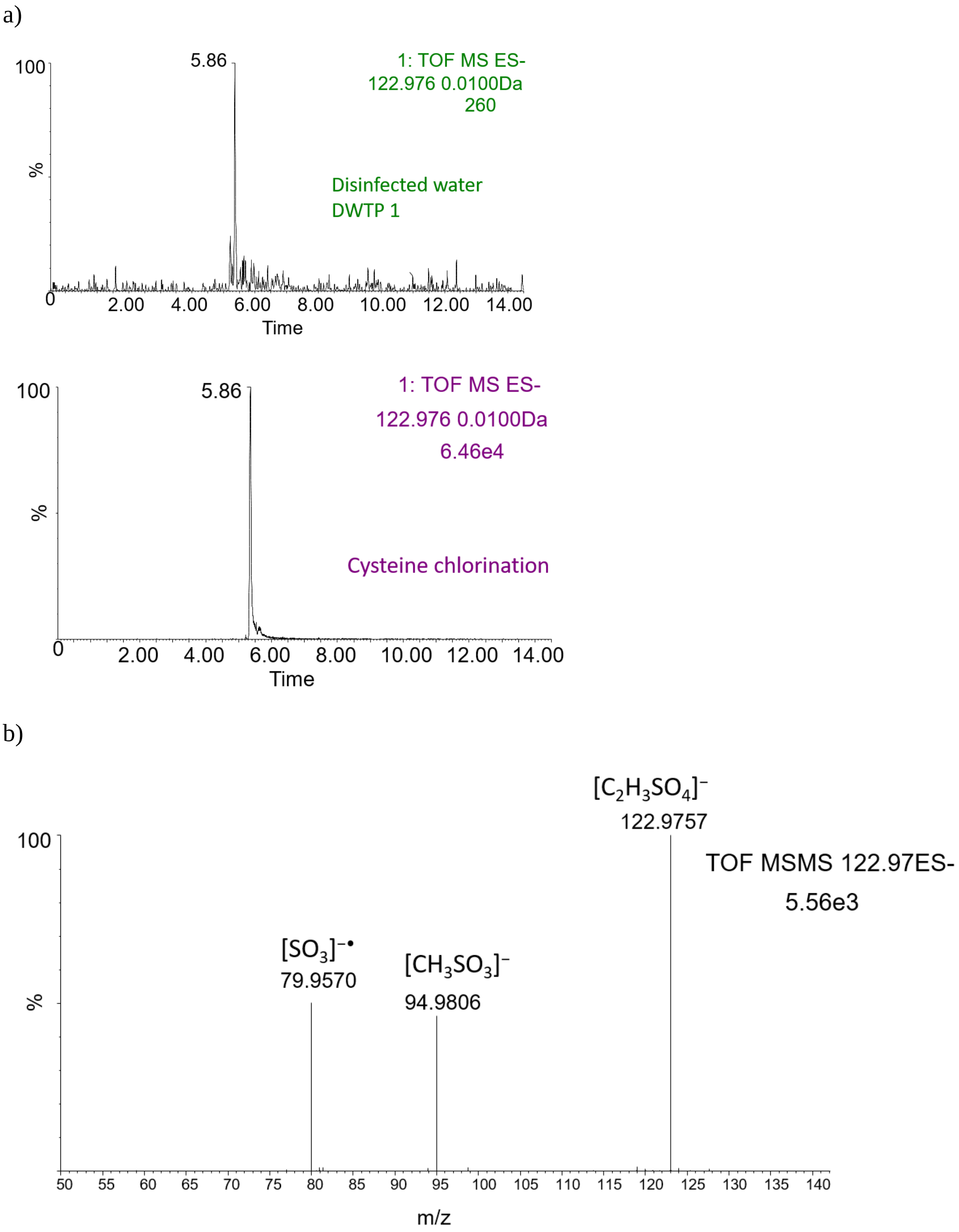

Figure S21. a) The extracted ion chromatogram of *m/z* 122.976 obtained during SFC-QTOF analysis of DWTP 1 and cysteine chlorination, and b) MS/MS spectrum of *m/z* 122.9757

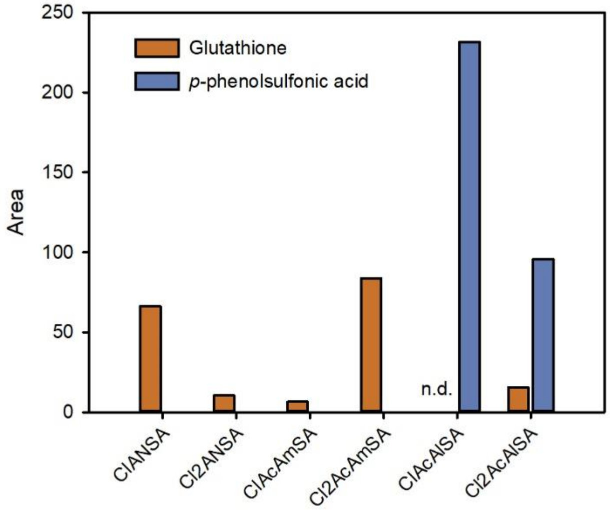

a)

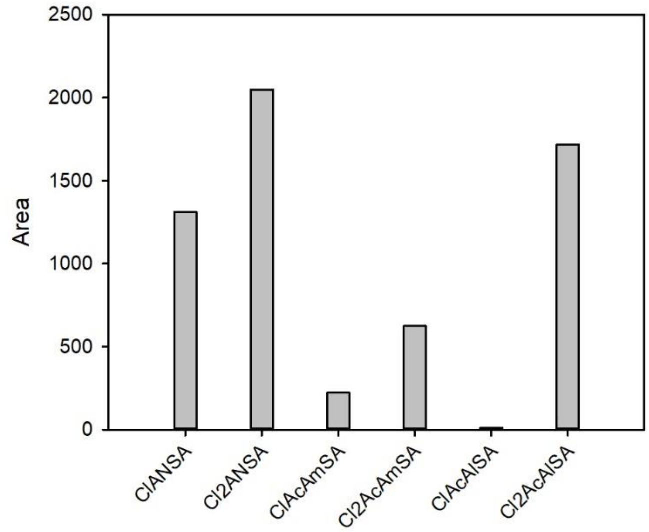

b)

Figure S22. Formation of novel sulfonic acid DBPs from the chlorination of glutathione (a), *p*-phenolsulfonic acid (a), and SRFA (b). Experimental conditions: 10 mM of phosphate buffer at pH 7; (a) Initial glutathione and *p*-phenolsulfonic acid: 250 µM, initial chlorine: 1.25 mM; (b) SRFA: 5 mg/L of DOC, initial chlorine: 5 mg/L as Cl_2. Chlorination of glutathione was conducted for 5 h and the sample was analysed by direct injection on SFC-QTOF without further enrichment. Chlorination of *p*-phenolsulfonic acid and SRFA were conducted for 48 h and the samples were analysed on SFC-QTOF after freeze-drying enrichment.

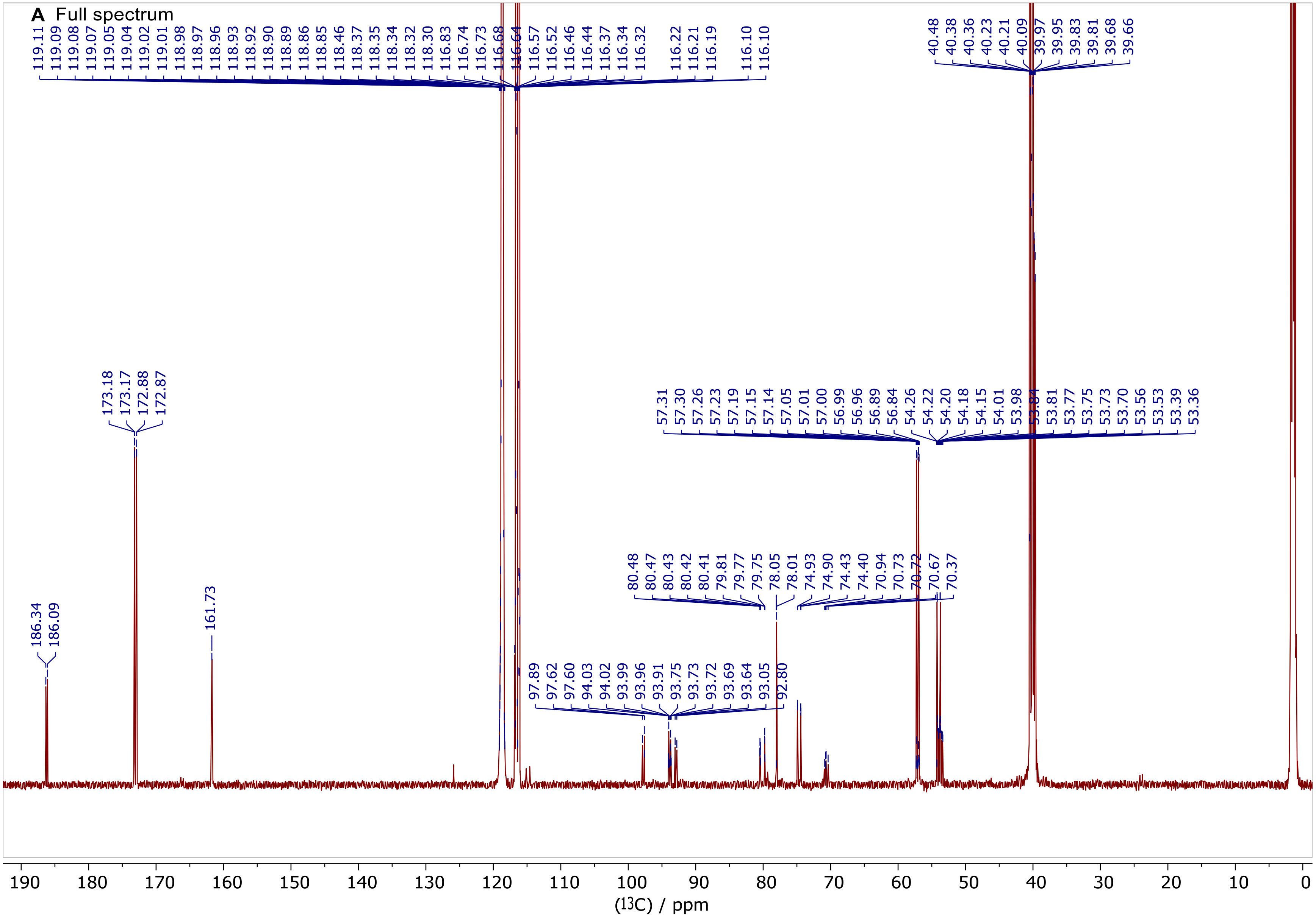
A Full spectrum
119.11
119.09
119.08
119.07
119.05
119.04
119.02
119.01
118.98
118.97
118.96
118.93
118.92
118.90
118.89
118.86
118.85
118.46
118.37
118.35
118.34
118.32
118.30
116.83
116.74
116.73
116.68
116.64
116.57
116.52
116.46
116.44
116.37
116.34
116.32
116.22
116.21
116.19
116.10
116.10
40.48
40.38
40.36
40.23
40.21
40.09
39.97
39.95
39.83
39.81
39.68
39.66
173.18
173.17
172.88
172.87
57.31
57.30
57.26
57.23
57.19
57.15
57.14
57.05
57.01
57.00
56.99
56.96
56.89
56.84
54.26
54.22
54.20
54.18
54.15
54.01
53.98
53.84
53.81
53.77
53.75
53.73
53.70
53.56
53.53
53.39
53.36
80.48
80.47
80.43
80.42
80.41
79.81
79.77
79.75
78.05
78.01
74.93
74.90
74.43
74.40
70.94
70.73
70.72
70.67
70.37
186.34
186.09
161.73
97.89
97.62
97.60
94.03
94.02
93.99
93.96
93.91
93.75
93.73
93.72
93.69
93.64
93.05
92.80
190
180
170
160
150
140
130
120
110
100
90
80
70
60
50
40
30
20
10
0
(13C) / ppm

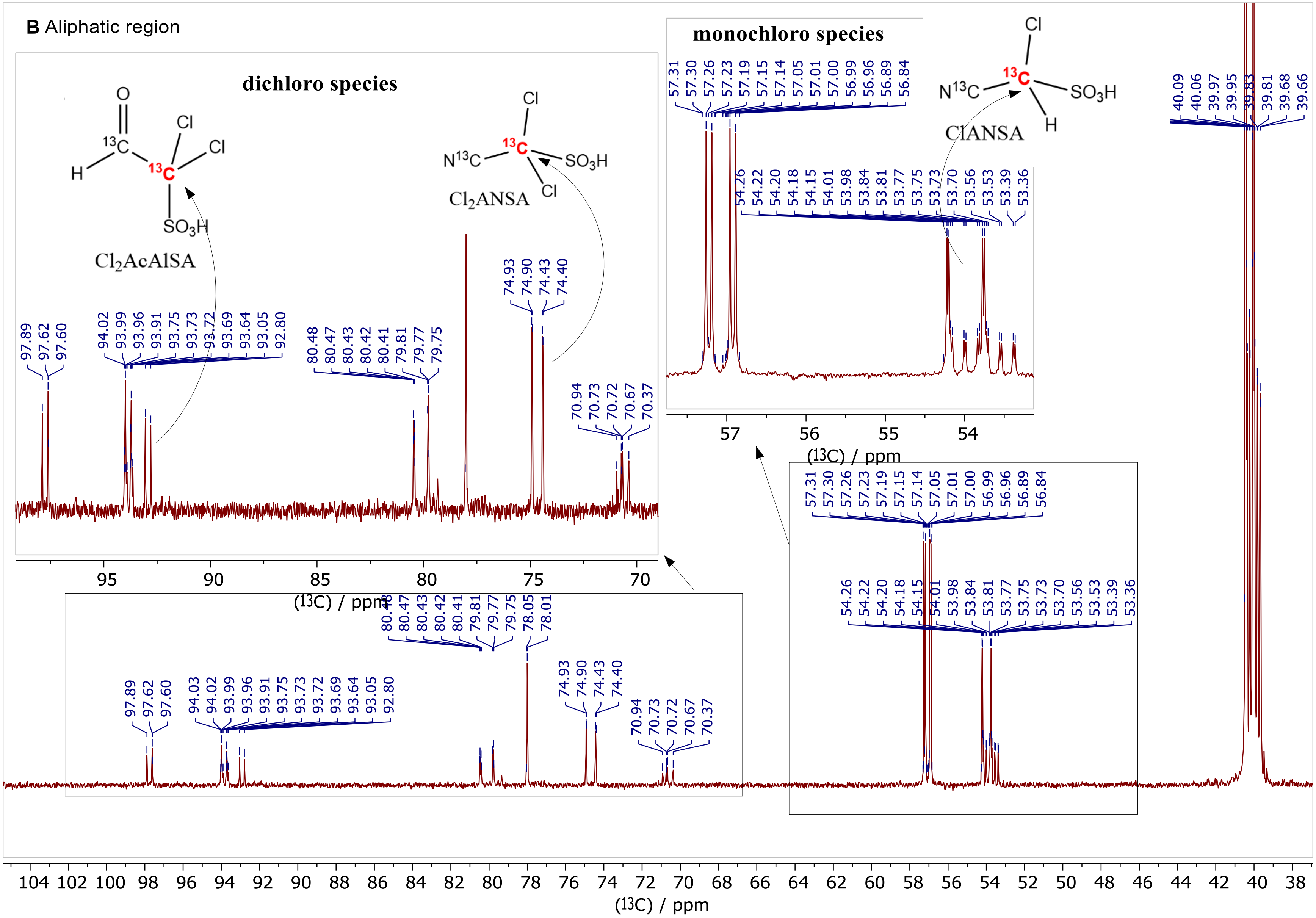

B Aliphatic region
dichloro species
Cl2AcAlSA
Cl2ANSA
monochloro species
ClANSA
97.89 97.62 97.60
94.03 94.02 93.99 93.96 93.91 93.75 93.73 93.72 93.69 93.64 93.05 92.80
80.48 80.47 80.43 80.42 80.41 79.81 79.77 79.75
78.05 78.01
74.93 74.90 74.43 74.40
70.94 70.73 70.72 70.67 70.37
57.31 57.30 57.26 57.23 57.19 57.15 57.14 57.05 57.01 57.00 56.99 56.96 56.89 56.84
54.26 54.22 54.20 54.18 54.15 54.01 53.98 53.84 53.81 53.77 53.75 53.73 53.70 53.56 53.53 53.39 53.36
40.09 40.06 39.97 39.95 39.83 39.81 39.68 39.66
(13C) / ppm

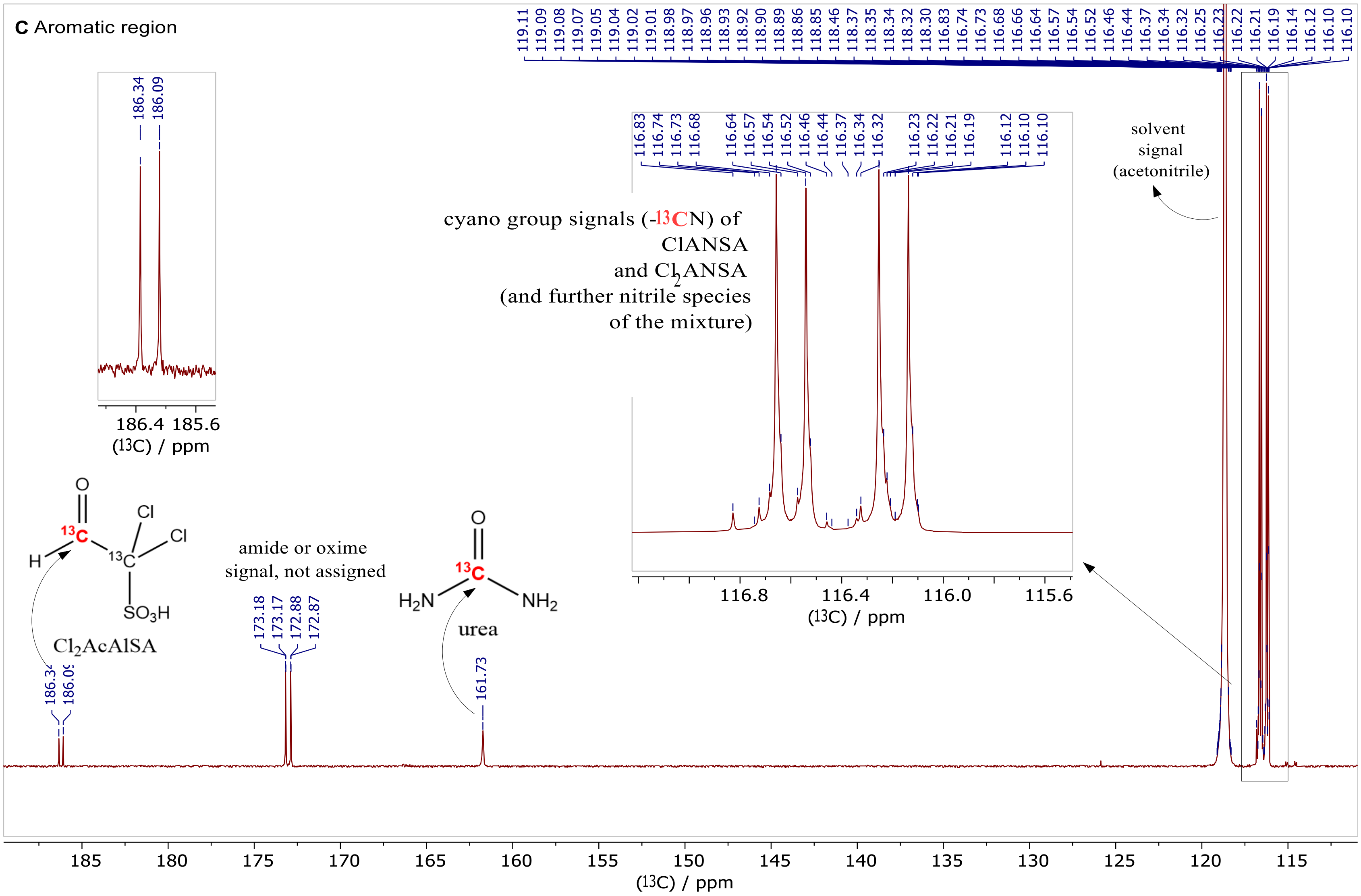
C Aromatic region
119.11 119.09 119.08 119.07 119.05 119.04 119.02 119.01 118.98 118.97 118.96 118.93 118.92 118.90 118.89 118.86 118.85 118.46 118.37 118.35 118.34 118.32 118.30 116.83 116.74 116.73 116.68 116.66 116.64 116.57 116.54 116.52 116.46 116.44 116.37 116.34 116.32 116.25 116.23 116.22 116.21 116.19 116.14 116.12 116.10 116.10
186.34 186.09
186.4 185.6
(13C) / ppm
116.83 116.74 116.73 116.68 116.64 116.57 116.54 116.52 116.46 116.44 116.37 116.34 116.32 116.23 116.22 116.21 116.19 116.12 116.10 116.10
cyano group signals (-13CN) of ClANSA and Cl2ANSA (and further nitrile species of the mixture)
116.8 116.4 116.0 115.6
(13C) / ppm
solvent signal (acetonitrile)
Cl2AcAlSA
amide or oxime signal, not assigned
173.18 173.17 172.88 172.87
urea
161.73
186.34 186.09
185 180 175 170 165 160 155 150 145 140 135 130 125 120 115
(13C) / ppm

D ^{1}H NMR: full spectrum and aromatic region

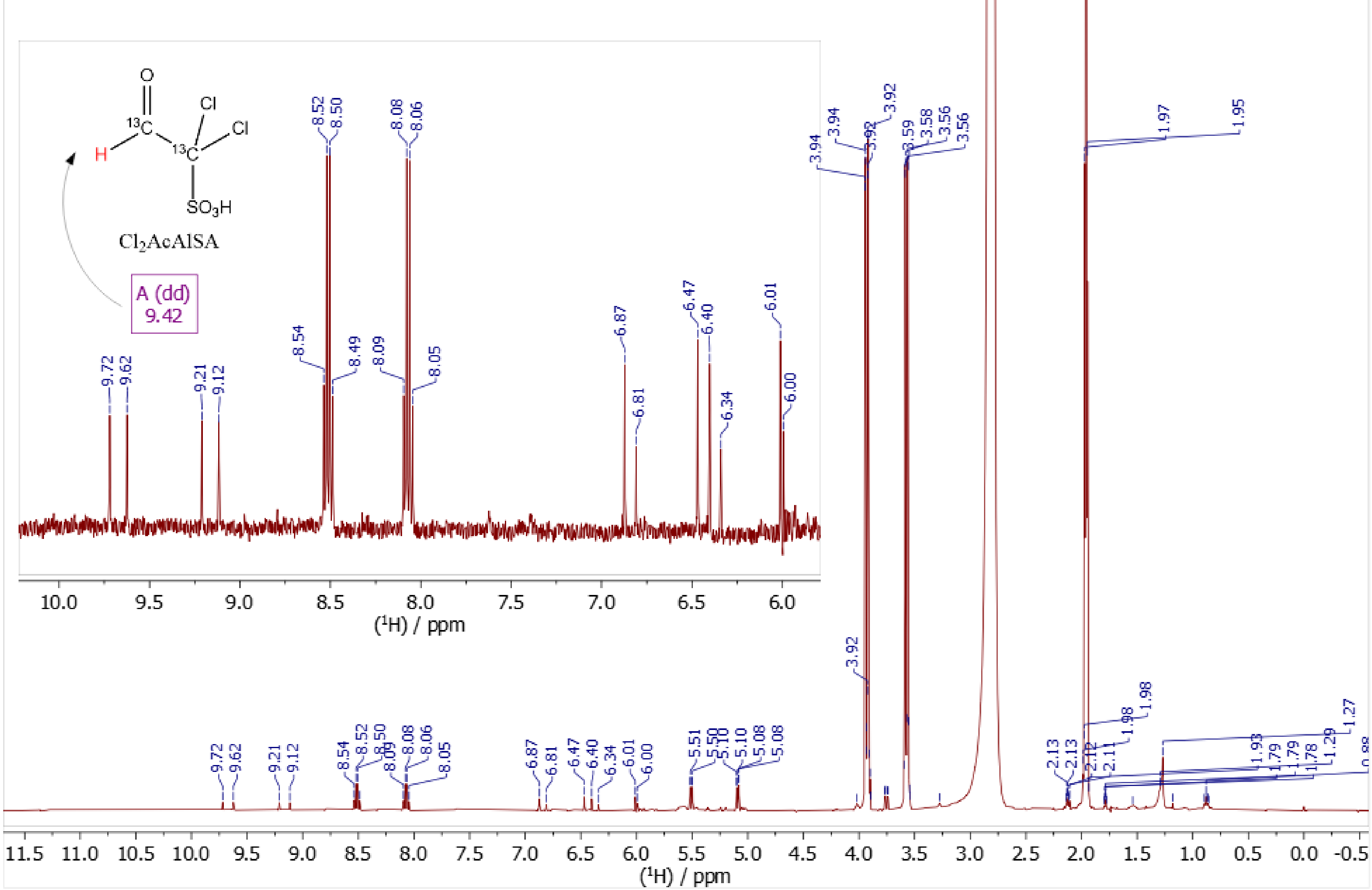

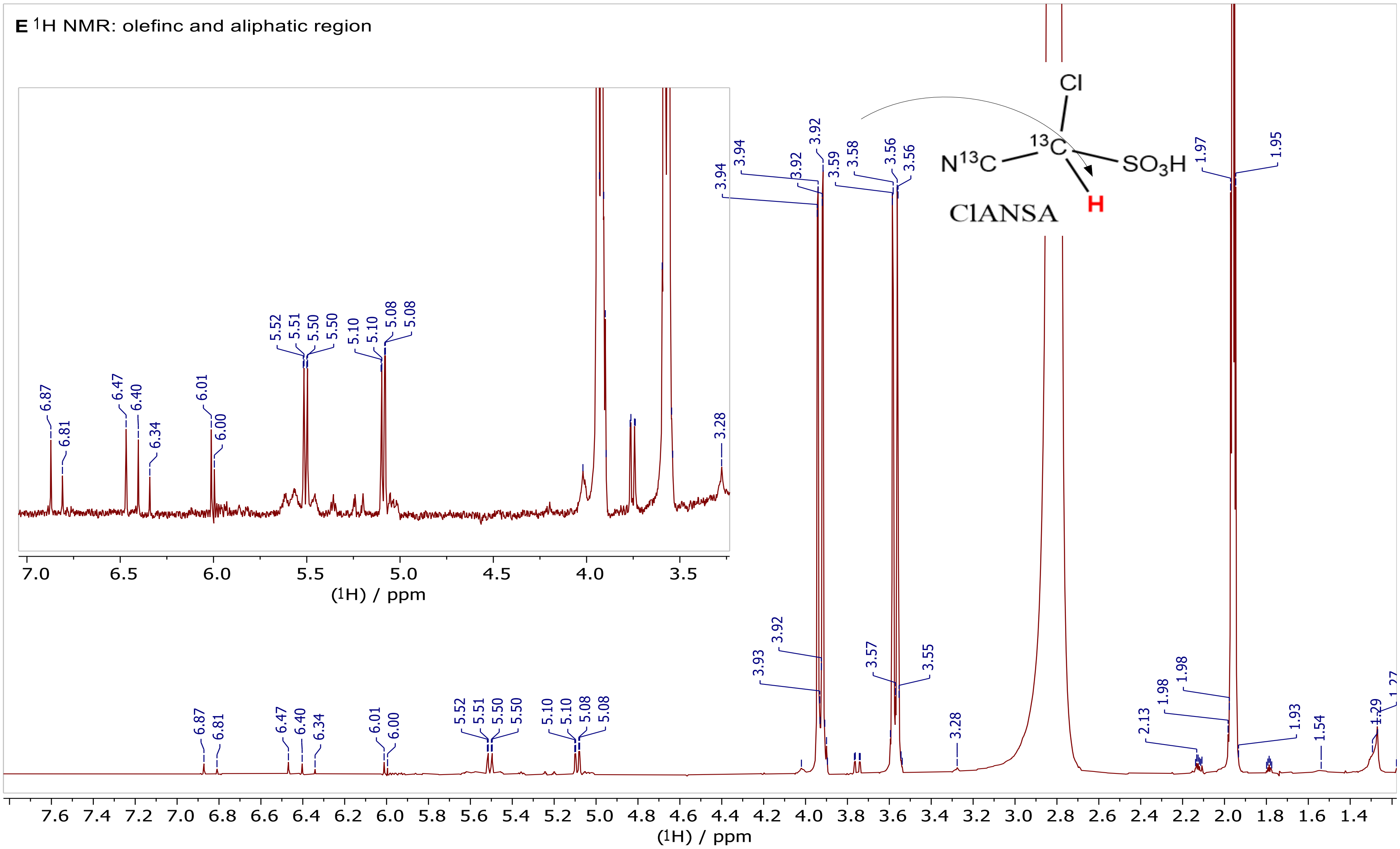

E ¹H NMR: olefinc and aliphatic region
ClANSA
(1H) / ppm

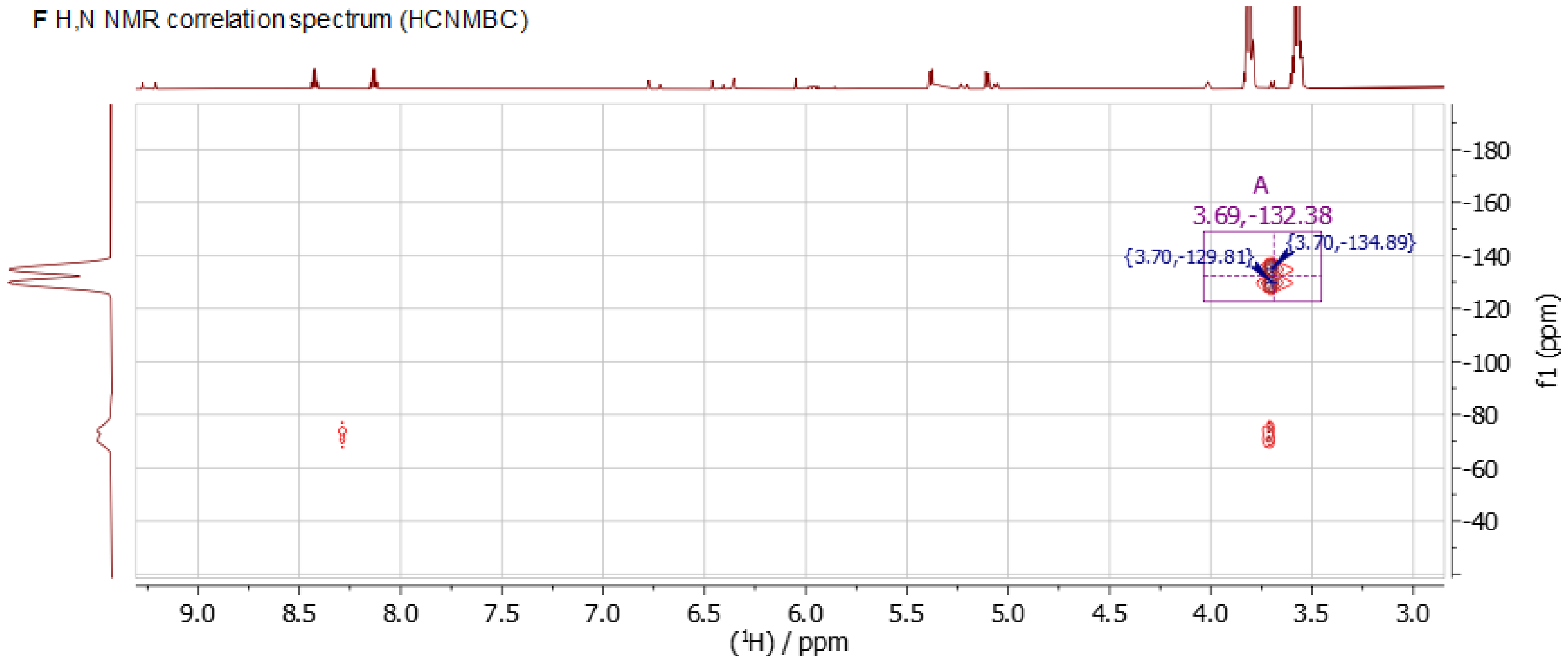

Figure S23. $^{13}C\{^{1}H\}$, ^{1}H NMR and H,N 2D correlation spectra of the DBP mixture including urea-^{13}C as internal standard: A) Full ^{13}C spectrum; B) aliphatic region of ^{13}C spectrum, C) aromatic region of ^{13}C spectrum, D) + E) ^{1}H spectra with aromatic and aliphatic region, respectively, F) H,N-2D HCNMB correlation spectrum. Nearly all ^{13}C signals show a signal splitting due to spin-spin-coupling to nuclei like further ^{13}C close by or ^{15}N from nitrile or amide/imine groups within the molecules. However, the ^{13}C spectrum has been acquired under proton decoupling. Description of Figure S23 is given in Text S1.

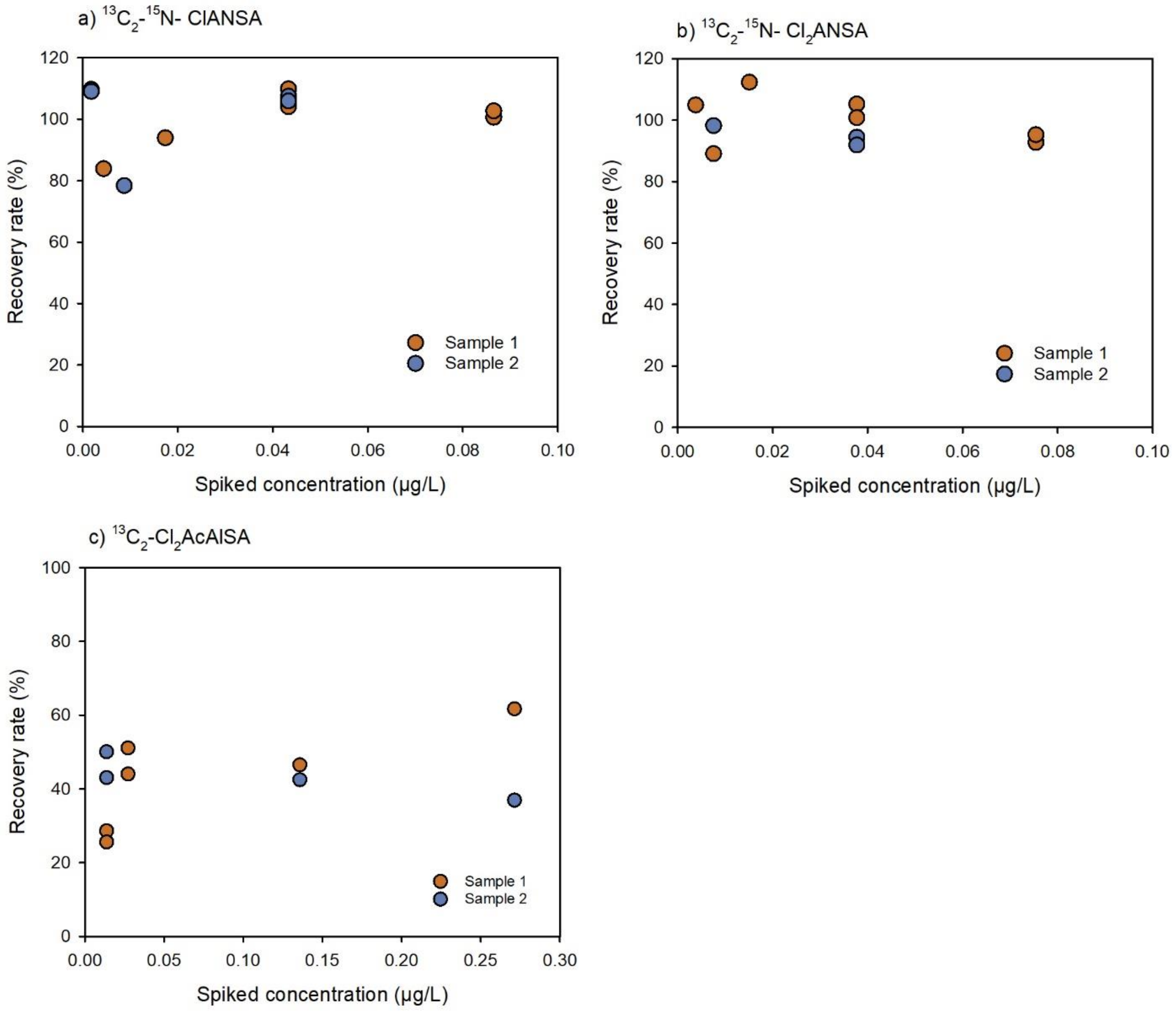

Figure S24. The apparent recovery rates of $^{13}C_2$-^{15}N -ClANSA (a), $^{13}C_2$-^{15}N -Cl_2ANSA (b), and $^{13}C_2$-Cl_2AcAlSA (c) during freeze-drying followed by SFC-QTOF analysis. Compounds (as DBP mixture produced from cysteine chlorination) were spiked into the 40 mL of aliquots of non-disinfected water samples from DWTP 1 (sample 1) and DWTP 2 (sample 2) at various concentrations.

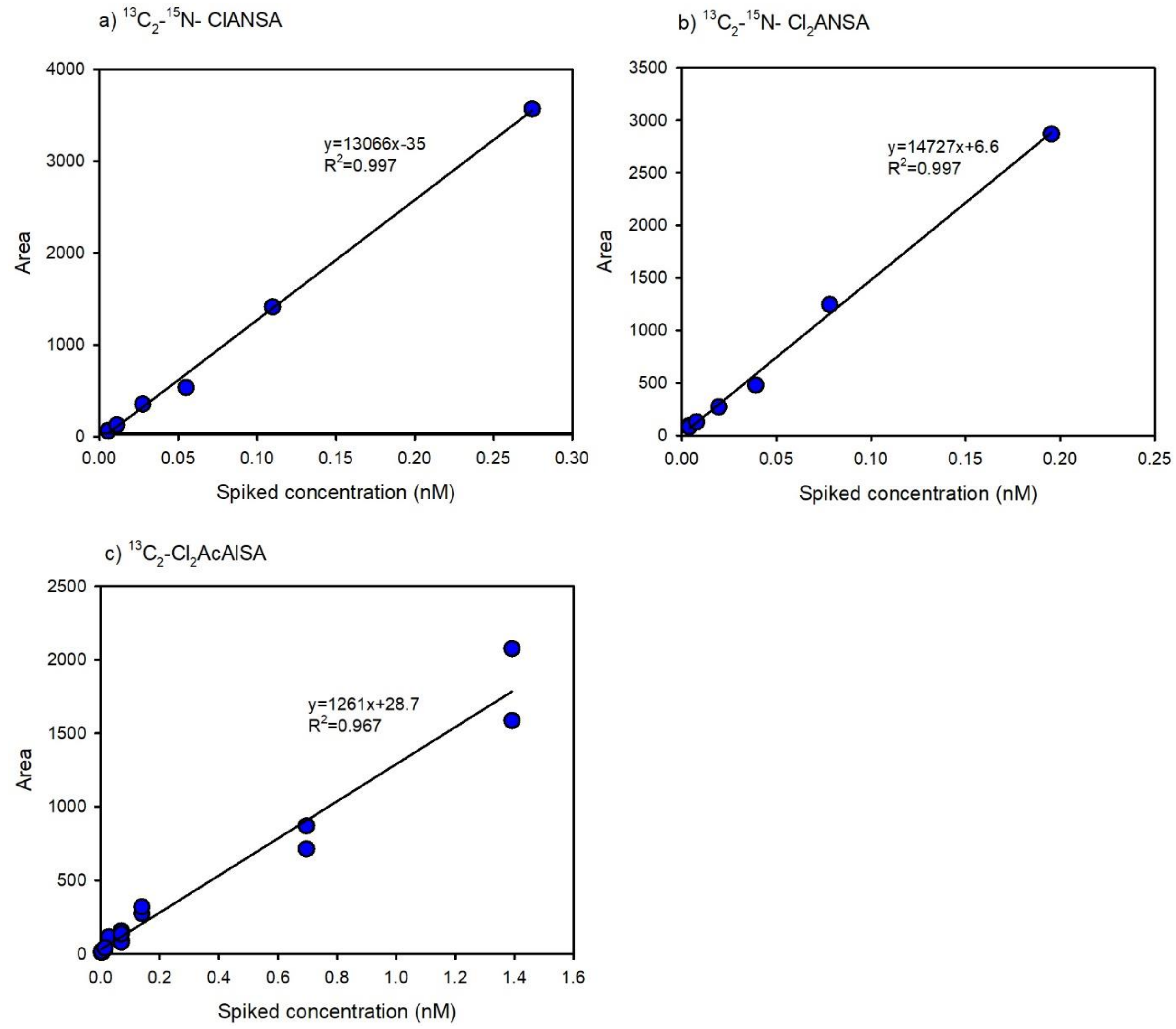

Figure S25. The processed calibration curves of $^{13}C_2$-^{15}N - ClANSA (a), $^{13}C_2$-^{15}N - Cl_2ANSA (b), and $^{13}C_2$- Cl_2AcAlSA (c). Compounds (as DBP mixture produced from cysteine chlorination) were spiked into the 40 mL aliquots of the non-disinfected water samples from DWTP 1 and 2, and analysed using SFC-QTOF after freeze-drying enrichment.

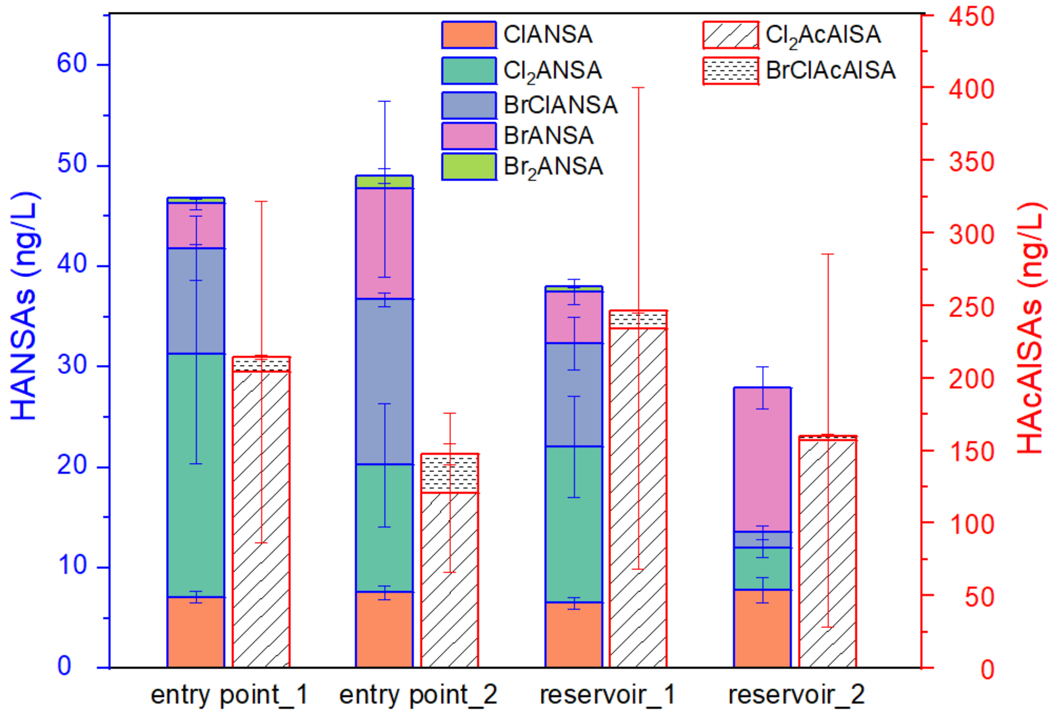

Figure S26. The estimated concentrations of HANSAs and HAcAlSAs (right Y-axis) at the two entry points to the drinking water distribution system as well as in the two storage reservoirs within this distribution system of a city in Hungary. The error bars represent the standard deviation of concentrations obtained during different sampling dates.

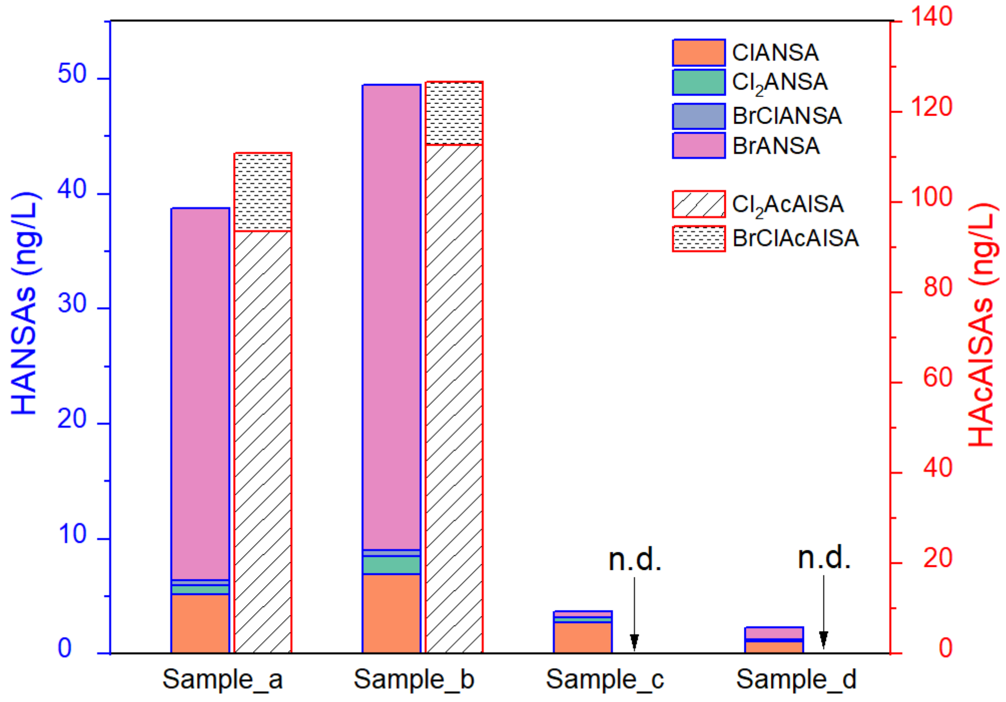

Figure S27. The estimated concentrations of HANSAs and HAcAlSAs (right Y-axis) in tap water samples collected from a city. “Sample_a” and “Sample _b” were collected from area with the application of booster chlorination in distribution system, whereas no booster chlorination was applied in the area of “Sample_c” and “Sample_d”. n.d. means non-detectable.

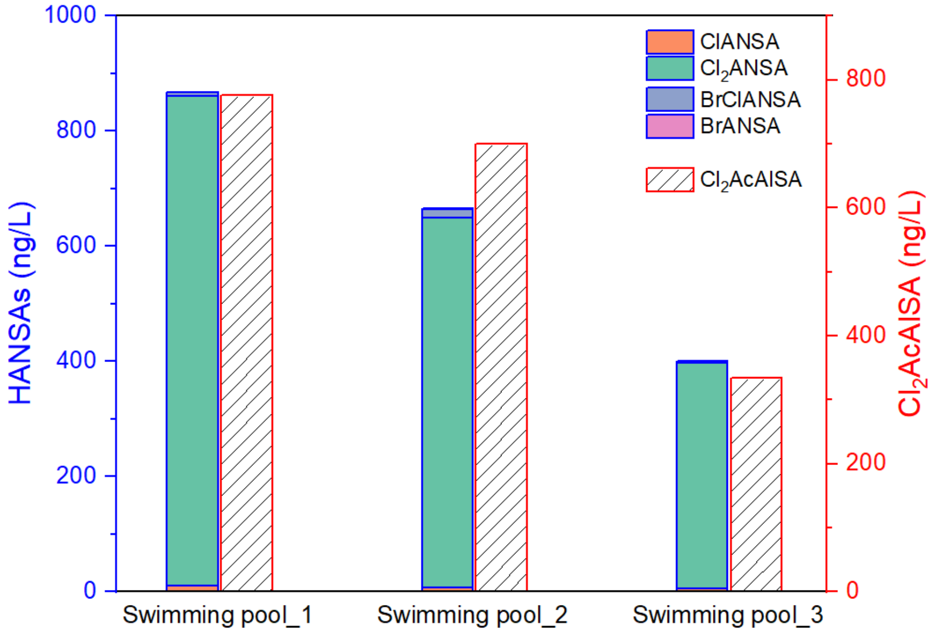

Figure S28. The estimated concentrations of HANSAs and Cl_2AcAlSA (right Y-axis) in three public swimming pools in Germany.
